# Plankton population dynamics and methylmercury bioaccumulation in the pelagic food web of mine-impacted surface water reservoirs

**DOI:** 10.1007/s10750-022-05018-0

**Published:** 2022-10-01

**Authors:** Mark Seelos, Marc Beutel, Stephen McCord, Sora Kim, Katie Vigil

**Affiliations:** 1 Environmental Systems Graduate Program, University of California Merced, Merced, CA 95343 USA; 2 Valley Water, San Jose, CA 95118 USA; 3 McCord Environmental, Inc, Davis, CA 95616 USA; 4 Department of Life and Environmental Sciences, University of California Merced, Merced, CA 95343 USA; 5 Department of Global Environmental Health, School of Public Health and Tropical Medicine, Tulane University, New Orleans, LA 70112 USA

**Keywords:** Methylmercury, Bioaccumulation, Phytoplankton, Zooplankton, Stable isotopes

## Abstract

Thermal stratification of reservoirs can lead to anaerobic conditions that facilitate the microbial conversion of mercury (Hg) to neurotoxic and bioaccumulative methylmercury (MeHg). But MeHg production is just the first step in a complex set of processes that affect MeHg in fish. Of particular relevance is uptake into suspended particulate matter (SPM) and zooplankton at the base of the pelagic food web. We assessed plankton dynamics and Hg uptake into the pelagic food web of four Hg-impaired California water reservoirs. Combining water chemistry, plankton taxonomy, and stable carbon (C) and nitrogen (N) isotope values of SPM and zooplankton samples, we investigated differences among the reservoirs that may contribute to differing patterns in MeHg bioaccumulation. Methylmercury accumulated in SPM during the spring and summer seasons. Percent MeHg (MeHg/Hg*100%) in SPM was negatively associated with δ^15^N values, suggesting that “fresh” algal biomass could support the production and bioaccumulation of MeHg. Zooplankton δ^13^C values were correlated with SPM δ^13^C values in the epilimnion, suggesting that zooplankton primarily feed in surface waters. However, zooplankton MeHg was poorly associated with MeHg in SPM. Our results demonstrate seasonal patterns in biological MeHg uptake and how multiple data sources can help constrain the drivers of MeHg bioaccumulation.

**Supplementary Information:**

The online version contains supplementary material available at 10.1007/s10750-022-05018-0.

## Introduction

Mercury (Hg) is a pollutant of concern in lakes and reservoirs, particularly in systems that experience oxic–anoxic cycling at the sediment–water interface or in the water column (Branfireun et al., 2020). Natural or anthropogenic Hg in the atmosphere can be deposited in catchments through wet or dry deposition (Driscoll et al., 2013). This atmospheric Hg, along with Hg present in soils from geologic and mining sources, can be transported into aquatic systems with runoff (Hsu-Kim et al., 2018). Under reducing conditions, anaerobic microorganisms convert inorganic Hg into neurotoxic methylmercury (MeHg), which bioaccumulates in aquatic ecosystems (Bigham et al., 2017). Methylmercury concentrates in suspended particulate matter (SPM), which serves as the main source of MeHg to the pelagic food web (Ogorek et al., 2021). Further accumulating in zooplankton and their predators through dietary intake, MeHg concentrations in fish can exceed aqueous concentrations by 7 orders of magnitude (Ogorek et al., 2021). Ingestion of fish contaminated with MeHg can cause a range of neurological, reproductive, and cardiovascular defects in humans (Hong et al., 2012), and behavioral, neurochemical, hormonal, and reproductive changes in mammals, birds, and fish (Scheuhammer et al., 2007).

Despite the widely accepted conceptual model of MeHg diffusion into the water column followed by concentration in SPM and accumulation in the pelagic food web, the drivers that determine the degree of MeHg bioaccumulation are poorly understood. A survey of Hg concentrations in freshwater fish throughout the western United States and Canada showed that fish Hg was not correlated with sediment Hg, and only weakly correlated with sediment MeHg (Eagles-Smith et al., 2016). A 2019 meta-analysis of 32 journal articles representing 22 sites worldwide showed that water column MeHg concentrations did not predict MeHg concentrations in biota (Wu et al., 2019). These surprising results are due to the multitude of chemical, biological, and ecological processes that govern MeHg uptake and bioaccumulation.

Net production of MeHg occurs when the rate of Hg methylation exceeds the rate of MeHg demethylation, both of which can be influenced by biological and chemical factors. Many of the factors influencing the net production and bioaccumulation of MeHg in aquatic systems appear contradictory. For example, eutrophic systems with abundant nutrients, dissolved organic matter (DOM), and oxic–anoxic cycling can support high rates of net MeHg production (Bravo et al., 2017; Eckley et al., 2017; Herrero Ortega et al., 2018). On the other hand, phytoplankton biomass can decrease MeHg uptake into the base of the pelagic food web through “bloom dilution” and promote rapid, efficient growth in fish and zooplankton that can further dilute MeHg in biota (Pickhardt et al., 2002; Karimi et al., 2007; Ward et al., 2010). Dissolved organic matter also plays a dual role in MeHg production and bioaccumulation, serving to stimulate microbial metabolism that enhances Hg methylation, while also decreasing the bioavailability of inorganic Hg(II) to methylating microbes, promoting MeHg photodegradation, and attenuating uptake of MeHg into the food web (Ravichandran, 2004; Luengen et al., 2012; Graham et al., 2013; Chiasson-Gould et al., 2014; Qian et al., 2014). Food web structure can affect the degree of MeHg bioaccumulation. Many studies show elevated MeHg bioaccumulation with increased food web length (Cabana et al., 1994; Ouédraogo et al., 2015; Thomas et al., 2016). Uptake of MeHg can vary considerably among different algal species, highlighting the importance of algal assemblages in controlling MeHg bioconcentration in pelagic food webs (Lee and Fisher, 2016). Finally, the diets and grazing strategies of fish and zooplankton can influence their MeHg concentration. Grazing depth and diet (e.g., bacteria vs. algae) can influence MeHg concentrations in zooplankton (Kainz and Mazumder, 2005; Hannides et al., 2013). Pelagic fish commonly contain significantly higher MeHg concentrations than their benthic counterparts (Matthews and Mazumder, 2005; Ouédraogo et al., 2015). Foraging strategy may change over a species’ lifecycle or because of an ecosystem perturbation, causing differences in MeHg bioaccumulation over time (Eagles-Smith et al., 2008).

Stable isotope values of carbon (δ^13^C) and nitrogen (δ^15^N) in SPM and zooplankton are valuable tools that can help ascertain the processes that drive differences in MeHg bioaccumulation between ecosystems. Carbon isotope values of SPM can elucidate the origin (i.e., autochthonous vs. allochthonous) of the detritus that forms the base of the planktonic food web, a factor known to affect MeHg production and bioaccumulation (Wang and Druffel, 2001; Bravo et al., 2017). Nitrogen isotope values of SPM can help identify the source of N to primary producers, an important aspect of energy flow in aquatic systems (Miyake and Wada, 1967). Carbon and N isotope values of SPM and zooplankton can help identify trophic linkages that facilitate the trophic transfer of MeHg to higher level organisms (Stewart et al., 2008). Finally, C and N isotope values in zooplankton can help identify the relative abundance of predatory and grazer zooplankton species, which can affect the degree of MeHg bioaccumulation within the planktonic food web (Post, 2002).

Patterns of MeHg bioaccumulation can be site-specific, necessitating focused studies in polluted systems to identify major drivers. In this field study, we assessed factors governing the uptake of Hg and MeHg into SPM and zooplankton in four Hg-impaired surface water reservoirs over four seasons from 2019 to 2021. We focused on uptake into the base of the pelagic food web because this is known to be the key step that controls MeHg concentration in fish (Lehnherr, 2014; Wu et al., 2019; Ogorek et al., 2021). Combining water chemistry data, algal and zooplankton taxonomic composition, and stable C and N isotope values of SPM and zooplankton samples, we investigated key differences between the reservoirs that may contribute to discrepancies in Hg and MeHg bioaccumulation. Specifically, our research questions were: (1) what are the chemical and biological similarities and differences between the study reservoirs? (2) does the abundance or structure of phytoplankton and zooplankton populations affect the degree of MeHg uptake into the food web? and (3) what factors contribute to enhanced MeHg uptake in SPM or zooplankton? Our hypotheses were that MeHg bioaccumulation would be greater in reservoirs with lower total plankton abundance, higher relative cyanobacteria abundance, and higher autochthonous organic matter concentrations. Our study shed light on some of the ways that plankton dynamics affect MeHg bioaccumulation, showing the seasonal patterns of MeHg uptake, and the importance of the composition of SMP and zooplankton grazing patterns.

## Site description and methods

### Site description

Almaden (AR), Calero (CR), and Guadalupe (GR) reservoirs are small, mesotrophic water storage reservoirs located in the upper Guadalupe River Watershed (San Jose, CA, USA), draining to South San Francisco (SF) Bay (Fig. S1). Almaden Reservoir and GR are contaminated by Hg-laden runoff from the former New Almaden Mining District, North America’s largest and most productive historical Hg mining district. Despite extensive remediation projects to contain and remove contaminated sediments, the New Almaden Mining District remains a major source of Hg (120 kg/yr) to the SF Bay (McKee et al., 2017). Calero Reservoir is in an adjacent subwatershed but has received contaminated water and sediments from AR through the Almaden-Calero Canal (Fig. S1). Stevens Creek Reservoir (SCR) has no known Hg mines in its watershed. The Hg source to SCR is assumed to be a combination of local and global atmospheric deposition (Rothenberg et al., 2010). All four reservoirs primarily receive inflow during California’s wet season (October–April), but CR’s capacity is maintained year-round through water imports from the Sacramento-San Joaquin Delta. The variable characteristics of local geology, land use, water source, and reservoir management are reflected in the range of Hg concentrations and primary productivity of each reservoir (Table S1).

Fish in each reservoir exceed regulatory targets for Hg (SFBRWQCB, 2008). Total mercury in muscle tissue of 35-cm length-standardized largemouth bass [*Micropterus salmoides* (Lacépède, 1802)] ranges from 0.75 mg/kg (wet weight) in SCR to 4.9 mg/kg in GR (Seelos et al., 2021). Valley Water (San Jose, CA) installed line-diffuser hypolimnetic oxygenation systems (HOSs) in each reservoir to increase dissolved oxygen (DO) concentrations at the sediment–water interface and curtail Hg methylation (McCord et al., 2016). Hg concentrations 100 mm largemouth bass have declined significantly in GR and SCR since HOS, by about 55% and 37%, respectively (Seelos et al., 2021). The HOSs were operated intermittently during the study period (Fig. S2).

### Field methods

We completed four seasonal monitoring events (Summer 2019, Winter 2020, Fall 2020, Spring 2021) in each reservoir to capture the seasonal and interannual variability in food web structure and Hg dynamics. Data were collected at the deepest portions of the reservoir, above the bottom-release outlet structures, to target the pelagic food web. In addition, Valley Water collected water quality profiles at these locations monthly to bi-monthly (with monitoring gaps in 2020 due to the COVID-19 pandemic).

#### Water sampling

Using Hydrolab DS5 sondes, we measured vertical profiles of temperature, DO, pH, oxidation–reduction potential (ORP), specific conductivity, chlorophyll *a*, and phycocyanin from the reservoir surface to the bottom at ≤ 1 m sampling interval. Grab samples were collected at discrete depths using a Wildco ® horizontal Van Dorn trace metal sampler and dispensed using ultraclean handling methods (USEPA, 1996). Surface (1–2 m depth) and bottom (1–7 m from bottom) water samples were dispensed into proper containers for analysis of total Hg, total MeHg, sulfate, and ammonia. 500 mL of surface and bottom water were collected into acid-washed teflon bottles for later filtration and filter-passing MeHg analysis (FP MeHg). Three additional samples were taken between the surface and bottom samples and analyzed for MeHg. 125 mL water samples were collected at the surface, middle, and bottom depths and dispensed into amber glass bottles for analysis of algal taxonomy. An additional 4 L of water was collected at each depth for the isolation of SPM. The reservoir storage volume and thermal structure of each reservoir varied at each sample collection, providing a range of chemical and biological conditions (Fig. S3). Water samples were placed on ice and transported to the laboratory for analysis.

#### Zooplankton sampling

Zooplankton samples were collected using an 80-µm nylon plankton net with a 0.5 m diameter opening (Aquatic Research Instruments). For taxonomic analysis, we collected a single vertical tow from 6 to 12 m depth (depending on reservoir stage) and washed all contents into a glass jar using deionized water. When the reservoirs were stratified, samples were collected from within the hypolimnion to the surface. The sample depth was recorded for use in zooplankton density calculations. Several additional vertical tows were made at the same sampling depth as the taxonomy sample to collect sufficient zooplankton biomass for Hg, MeHg, and stable isotope analysis. The zooplankton taxonomy and biomass samples were held on ice until transport to the laboratory for processing and analysis.

#### Fish assemblage surveys

Though we were unable to collect fish samples during the study period due to the COVID-19 pandemic, historical fish assemblages offer insight into potential top-down controls on plankton populations. Using boat electrofishing with four netters, fish assemblages were estimated by capturing fish observed during 15-min shoreline passes, then calculating the observed catch per minute (CPM) per species.

### Sample processing

#### Water samples

Immediately upon arrival at the laboratory, FP MeHg samples were filtered using sterile polystyrene filtration apparatuses and 0.45 μm polyethersulfone filters, then decanted into 250 mL fluorinated polyethylene bottles. Samples to be analyzed for Hg, MeHg, and FP MeHg were preserved with 0.5% 12 N HCl. Ammonia samples were preserved to pH < 2 with H_2_SO_4_. Sulfate samples were unfiltered and unpreserved. All water samples were kept refrigerated (4 °C) until analysis.

#### Algae and SPM samples

Microscope slides were prepared for analysis of algal taxonomy on the day of collection. A 1:100 dilution of 1% Lugol’s iodine was added to water samples designated for algal taxonomy. Thirty-two mL of each sample were filtered onto a membrane filter grid (Metricel Grid: 47 mm, 0.45 um). Using clean forceps, each filter was placed sample-side down on a glass microscope cover slip. A thin layer of clear pre-polymerized 2-hydroxypropyl methacrylate (HPMA) mounting resin was spread on top of the filtered algae membrane and cured at ≥ 35 °C. Mounted slides were labeled and stored in a slide box until taxonomic analysis.

SPM in water samples was concentrated on filters for analysis of Hg, MeHg, total suspended solids (TSS), and stable isotopes of C and N. For each SPM sample, four liters of reservoir water were prefiltered using a 150-um mesh to remove coarse particulates. Using a glass filtration apparatus, SPM was concentrated onto 0.45 μm glass fiber filters by pouring a known volume (~ 500 mL) of water through the filter until it clogged. The first subsample, used for analysis of TSS and stable C and N isotopes, was immediately transferred to an incubator and dried at 60 °C until a constant mass was obtained. TSS concentration was measured gravimetrically as the filter mass change divided by the volume of water filtered. TSS was measured using a low temperature because the filter SPM was subsequently encapsulated for stable isotope analysis (see below). Two additional subsamples were produced for analysis of Hg and MeHg, with the SPM mass on the filter calculated as the volume of water poured through the filter multiplied by the TSS. Using forceps, concentrated SPM samples for Hg and MeHg analysis were placed into acid-washed amber glass vials and frozen until analysis.

Following gravimetric analysis, the filters used for determination of TSS were placed in a desiccator overnight with a beaker containing > 12 N fuming HCl for removal of trace carbonates from the SPM samples. Each filter was cut into thirds using ethanol-cleaned scissors. Each filter subsample was carefully rolled and encapsulated in 9 × 10 mm tin capsules, placed in a 48-well tray, and stored in a desiccator for stable C and N isotope analysis. Filter method blanks for Hg, MeHg, TSS, and stable isotope analysis were prepared using the procedures described above with deionized water instead of reservoir water.

#### Zooplankton samples

Zooplankton samples for taxonomic analysis were preserved with a 1:100 dilution of 1% Lugol’s iodine, stored in a refrigerator at 4 °C, and analyzed within 48 h of collection (see below). Concentrated zooplankton biomass samples were triple rinsed by dilution to 2 L using deionized water, followed by filtration onto a 80-μm mesh. Rinsed zooplankton biomass samples were placed onto aluminum weighing dishes and dried in an incubator at 60 °C until a constant mass was obtained. Using a clean mortar and pestle, samples were homogenized into a fine powder. Triplicate subsamples of ~ 1 mg were encapsulated in 5 × 9 mm tin capsules, placed in a 96-well tray, and stored in a desiccator for stable C and N isotope analysis. The remaining homogenate was divided in half and transferred to separate acid-washed amber glass vials for Hg and MeHg analysis. Samples were frozen until analysis.

### Analytical methods

#### Taxonomic analysis

Algae were identified and quantified using a Zeiss Axio Imager A2 Microscope. The slides were first viewed using ×100 or ×200 magnification to ensure an even distribution of dominant taxa. All taxa present were identified to genus or species using various references and keys (e.g., Cox, 2006; Sime, 2004; Guiry and Guiry, 2022). For samples dominated by algae greater than 10–20 μm in the greatest axial or linear dimension (GALD), a minimum of 300 cells or natural units and 15 random fields were identified and counted at ×200 magnification (representing 1 mL). For samples that were dominated by algae less than 10–20 μm in GALD or containing fragile, difficult to identify taxa, a minimum of 400 natural units or cells and 25 fields were identified and counted at ×400 (representing 1 mL). Algae counts were made in triplicate and averaged. Algal cell densities were calculated for each taxa as the count multiplied by 32 (cells/mL). Cell densities were converted to biovolume based on literature approximations, and biomass assuming a cell density of 1 g/cm^3^ (Table S2).

Concentrated zooplankton biomass samples were diluted with tap water to 250 mL in a graduated cylinder. The sample was mixed thoroughly in the graduated cylinder with a stir bar to suspend the organisms randomly. Using a pipette, 1 mL subsample was quickly removed, transferred to a Sedgwick-Rafter cell, and covered with a microscope cover slip. The cell was placed under a compound microscope under a ×10 objective. All organisms were identified and counted in 5 subsamples. Zooplankton were identified to genus or species (Haney et al., 2013). Zooplankton density (count/m^3^) was calculated as (n*V_a_)/*V*_s_ where n is the number of zooplankton counted, *V*_a_ is the analyzed sample volume (250 mL), and *V*_s_ is the volume of water sampled using the zooplankton net (m^3^). Zooplankton biomass was estimated using literature measurements (Table S3).

#### Water Chemistry

Reservoir water samples were analyzed by Eurofins Scientific (Pleasanton, CA, USA). Total Hg was analyzed by oxidation, purge and trap, desorption, and cold-vapor atomic fluorescence spectrometry (CVAFS) (U.S. E.P.A., 2002). The method detection limit for total Hg is 0.2 ng/L. Total and FP MeHg were analyzed by distillation, aqueous ethylation, purge and trap, and CVAFS (U.S. E.P.A., 1998). The method detection limit for MeHg is 0.02 ng/L. Strict quality control standards were followed for trace-level Hg and MeHg analysis, including method blanks, matrix spikes/matrix spike duplicates (method requirement = 75–125% recovery), and check standards (method requirement = 77–123% recovery). Sulfate was analyzed by ion chromatography (U.S. E.P.A., 1993a, b). Ammonia was analyzed by semi-automated calorimetry (U.S. E.P.A., 1993a, b).

#### Hg and MeHg in solids

Solid-phase Hg and MeHg samples were analyzed by Eurofins Scientific (Sacramento, CA, USA). Total Hg in homogenized zooplankton and SPM (filters) were analyzed using a HNO_3_/H_2_SO_4_ digestion (3:7 ratio of 15.8 M HNO_3_ to 18 M H_2_SO_4_), followed by oxidation, purge and trap, desorption, and CVAFS (U.S. E.P.A., 2002). Methylmercury was extracted from solid samples using 25% KOH in methanol. Digestates were analyzed using distillation, aqueous ethylation, purge and trap, and CVAFS (U.S. E.P.A., 1998). Solid-phase Hg analysis followed strict quality control measures, including duplicates (acceptable relative percent difference = 30%), matrix spikes/matrix spike duplicates, and method blanks. Homogenized zooplankton were used for QA/QC samples (duplicates, matrix spikes) because extra sample was available. Adequate recovery (75–125%) was verified using certified reference material (TORT-3).

### Stable isotope analysis

Zooplankton samples were analyzed for bulk δ^15^N and δ^13^C by element analysis and isotope ratio mass spectrometry (EA-IRMS) at the Stable Isotope Ecosystem Laboratory of UC Merced (SIELO) and UC Davis Stable Isotope Facility (SIF). Twenty-four zooplankton samples were analyzed at the SIELO via a Costech EA coupled to a Thermo Fisher Delta V Plus via CONFLO IV, while 24 zooplankton samples were analyzed at UC Davis with a PDZ Europa ANCA-GSL elemental analyzer coupled to a DZ Europa 20–20 IRMS. One hundred forty-four SPM samples were analyzed at UC Davis using a similar method, but with an Elementar Vario EL Cube or Micro Cube elemental analyzer. Samples were analyzed in conjunction with standard reference materials (UC Davis: IAEA-600, USGS-40, USGS-41, USGS-41a, USGS-42, USGS-43, USGS-61, USGS-64, USGS-65; UC Merced: USGS-40, USGS-41) and internal standards of known isotopic composition for scale normalization, drift correction, and mass linearity correction. Isotope values were reported in permil (‰) difference relative to international standards Vienna Pee Dee Belemnite (VPDB, δ^13^C), and Earth’s atmosphere (Air, δ^15^N). The standard deviation of reference materials at the SIF was 0.05 ‰ for δ^13^C and 0.07 ‰ for δ^15^N (n = 176). The standard deviation of reference materials analyzed at SIELO was 0.05 ‰ for δ^13^C and 0.05 ‰ for δ^15^N (n = 32). At both laboratories, absolute accuracy for calibrated reference materials was < 0.1‰ for both δ^13^C and δ^15^N. Zooplankton δ^13^C values were mathematically corrected for lipid content (Syväranta and Rautio, 2010) to account for high carbon fractionation during lipid synthesis. Carbon isotope values in SPM were not lipid-corrected.

### Statistical analysis

All plotting and statistical analyses were performed using the R programming language. Ordination analysis performed via nonmetric multidimensional scaling (NMDS) was used to examine similarities in biological communities between reservoirs and to identify potential drivers. Using a nonmetric rank-order approach, NMDS attempts to reduce large multidimensional datasets (e.g., multiple biological assemblages or sets of water quality data) into fewer dimensions to identify underlying patterns and gradients. Datasets (e.g., measured biological assemblages) that plot closely to each other are relatively similar in structure. All NMDS analyses were performed with the Vegan package in R, using the Bray–Curtis Dissimilarity Index, autotransformation (Wisconsin double standardization), and a maximum of 100 random starts (Oksanen et al., 2017). Analyses yielding stress values > 0.15 were rejected.

## Results

### Water chemistry

Seasonal patterns in thermal stratification and water chemistry of the study reservoirs before and after HOS have been described in detail (McCord et al., 2016; Seelos et al., 2021). Briefly, each reservoir was thermally stratified beginning around March and continuing until fall turnover around October (Fig. S3). Prior to the installation of the HOSs, reservoir stratification corresponded with depletion of DO in the hypolimnia and establishment of reducing conditions. Hypolimnetic sulfate depletion coincided with elevated MeHg concentrations, which typically began to increase during the spring and peaked around August before declining with the establishment of low-ORP conditions. During HOS operation, MeHg concentrations in the hypolimnia of all reservoirs decreased dramatically, but MeHg in surface waters remained comparable to pre-HOS levels. Primary productivity, measured as chlorophyll *a* and phycocyanin concentrations, increased in surface waters during HOS operation, possibly the result of enhanced mixing of nutrient-rich bottom waters into the photic zone.

**Fig. 1:**
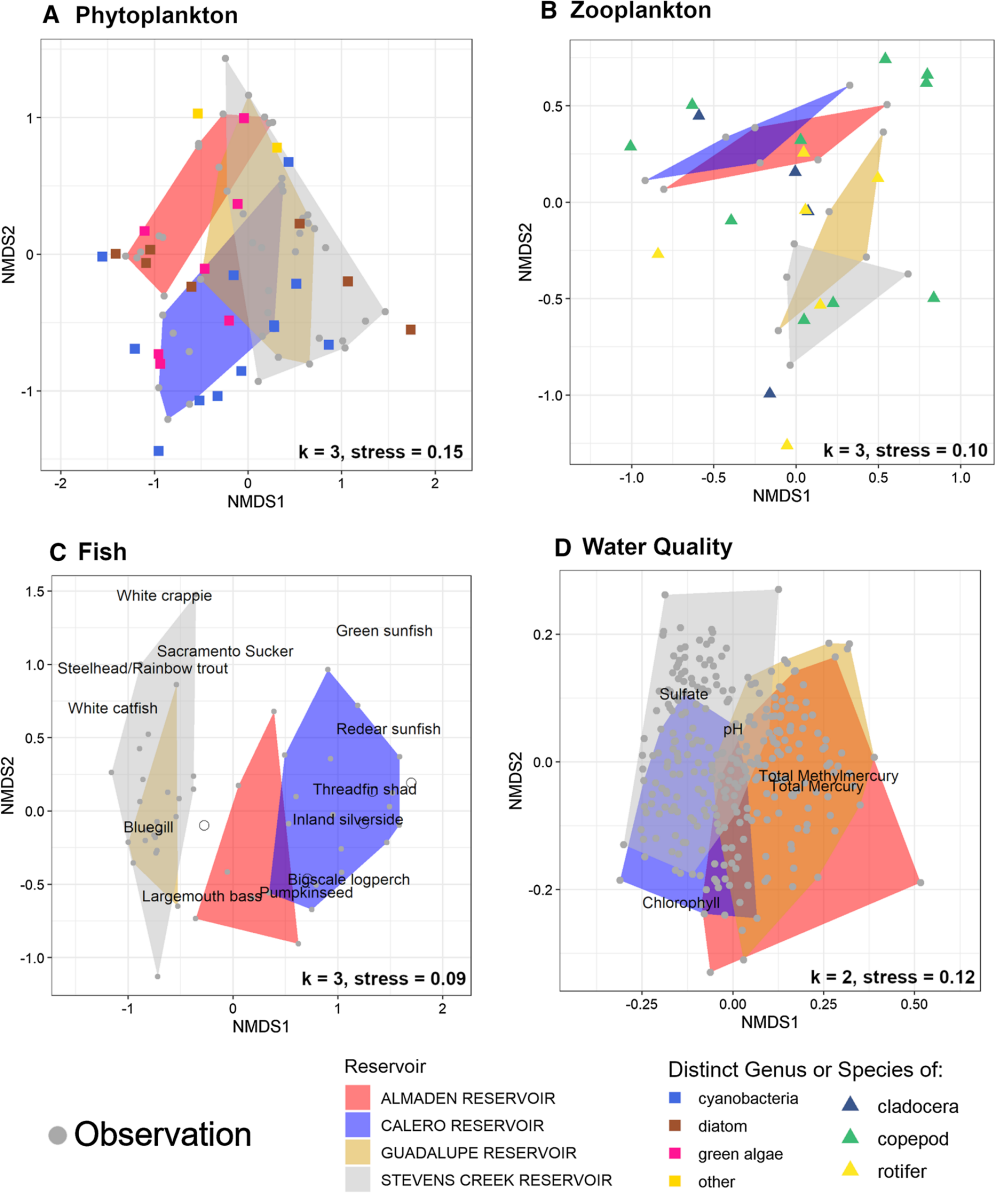
Nonmetric Multidimensional Scaling (NMDS) analysis of phytoplankton (**A**), zooplankton (**B**), fish (**C**), and water quality (**D**) data from four reservoirs. Analyses highlight ecological similarities between AR and CR, and between GR and SCR. Each grey point is an individual observation containing assemblage counts or a collection of water quality measurements in reduced dimensional space. The colored convex hulls enclose all individual observations made in each reservoir. In A and B, colored points represent a particular species or genus of the plankton group defined by the color (names not included to avoid cluttering, see legend for genus or species). In **C** and **D**, black text on plot denotes locations of relatively high values of the given species (**C**) or water quality parameter (**D**). The number of reduced dimensions (*k*) and Kruskal’s Stress (stress) are shown in the bottom-right of each plot

Despite similar limnological patterns and responses to HOS operation, the reservoirs had some notable differences post-HOS in the concentrations of key water quality parameters affecting MeHg production and bioaccumulation. Nonmetric multidimensional scaling (NMDS) analysis of key water quality parameters collected from surface waters of the four reservoirs from 2016 to 2021 showed distinct similarities between AR and GR, and between CR and SCR (Fig. 1d). Mercury and MeHg concentrations were notably higher in mine-impacted AR and GR, while CR and SCR had higher sulfate concentrations. Calero Reservoir had considerably higher chlorophyll *a* than the other three reservoirs.

### Biological assemblages

#### Phytoplankton assemblages

**Fig. 2:**
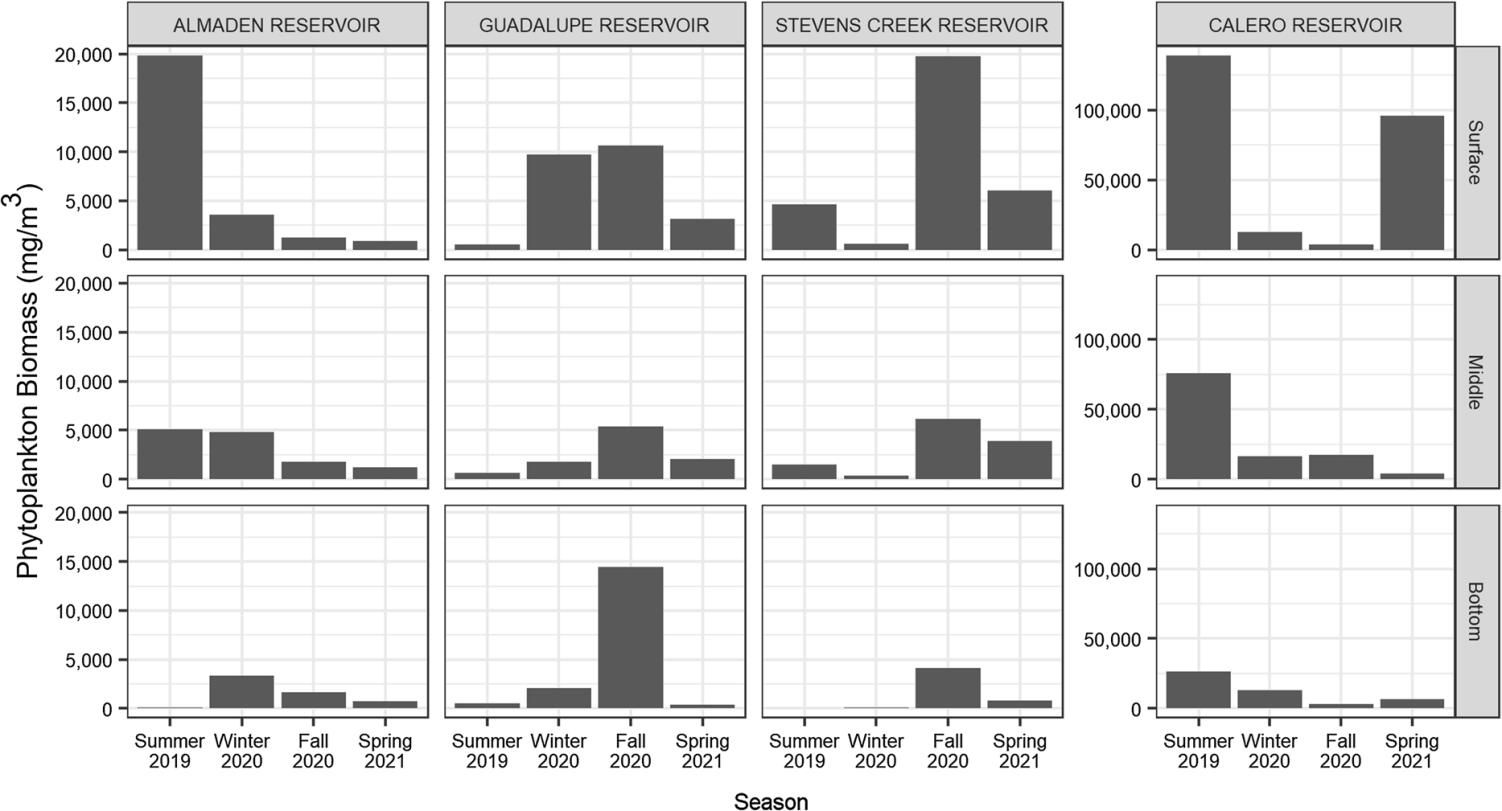
Total phytoplankton biomass concentrations measured in the surface, middle, and bottom sampling depths of each reservoir showing variable peaks and enhanced primary productivity in CR. Biomasses were calculated from algal counts using per-cell mass estimations are defined in Table S3

Each reservoir exhibited unique patterns of phytoplankton density and taxonomic distribution. Phytoplankton density was typically highest near the reservoir surfaces, decreasing with depth (Fig. 2). Calero Reservoir had notably higher phytoplankton density than the other reservoirs, peaking at 138 g/m^3^ in summer 2019 (Fig. 2). A second increase in phytoplankton density in CR surface water was measured during the spring 2021 sampling event. Unique to the reservoirs, the phytoplankton biomass in CR was comprised almost entirely of cyanobacteria year-round, apart from a notable fraction of green algae measured mid-water column during the fall 2020 sampling event (Fig. S4). Like CR, phytoplankton density in AR peaked in summer 2019, but the measured density was about 5 times lower in AR (Fig. 2). The phytoplankton assemblage at AR was the most diverse of the reservoirs, with the predominant biomass shifting seasonally between cyanobacteria (summer 2019), dinoflagellates (winter 2020), and diatoms (fall 2020) (Fig. S4). Guadalupe Reservoir and SCR had peak phytoplankton densities during the fall that were similar in magnitude, consisting predominantly of cyanobacteria (Fig. 2, Fig. S4). Shifts from cyanobacteria to green algae and dinoflagellates occurred in summer 2019 in GR and spring 2021 in SCR.

Ordination analysis of phytoplankton assemblages showed similarity in GR and SCR (Fig. 1a). Almaden Reservoir and CR had distinct assemblages with little community overlap with each other, but some overlap with GR and SCR (Fig. 1a). Two variables had significant associations with algal assemblages: pH (*p* = 0.01) and zooplankton concentration (*p* = 0.01) (Fig. S5a). Dinoflagellate and green algae species tended to be more abundant in samples with lower pH, while golden algae species were more prevalent at higher pH. Green algae and cyanobacteria abundance appeared to increase with zooplankton biomass.

#### Zooplankton assemblages

**Fig. 3:**
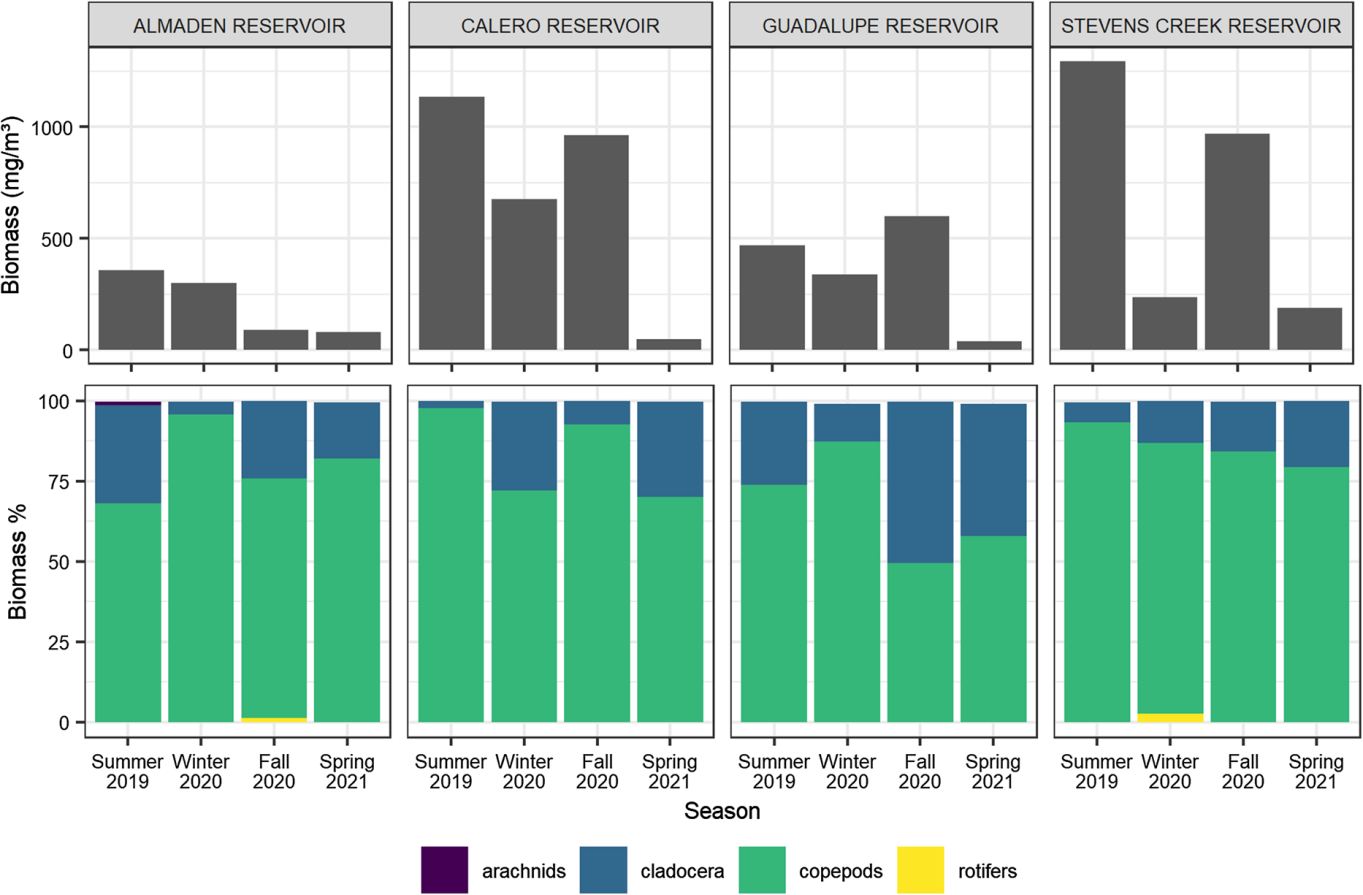
Total zooplankton biomass concentrations (top) and mass percentages per taxa (bottom) in each reservoir, showing relatively high density in CR and SCR and summer/fall peaks. Biomasses were calculated from zooplankton counts using per-organism mass estimations defined in Table S4

Seasonal patterns in zooplankton density were relatively consistent between the reservoirs. Calero Reservoir and SCR had comparable zooplankton densities, which were about twice as high as AR and GR. Zooplankton density peaked in summer 2019 and fall 2021 in all reservoirs except AR. Zooplankton density was relatively low in spring 2021 in all reservoirs. Zooplankton assemblages were comprised mainly of cladocerans, copepods, and rotifers, but copepods were dominant on a mass basis (Fig. S4, Fig. 3). There was no correlation between zooplankton biomass and algal biomass.

Ordination analysis of zooplankton assemblages showed considerable community overlap between AR and CR, and between GR and SCR (Fig. 1b). Algal biomass concentration was the only environmental variable that was significantly associated (p = 0.01) with zooplankton community composition (Fig. S5b). Copepod abundance appeared to increase with algae concentration while rotifer abundance decreased.

#### Fish assemblages

Total fish CPM (all species), a relative measure of overall fish abundance, was similar at CR, GR, and SCR, with a median of around 10 (Fig. S6). Total CPM at AR was notably lower than at the other reservoirs, with a median of 3. Fish assemblages varied considerably among reservoirs. The NMDS analysis showed considerable community overlap in GR and SCR (Fig. 1c). Fish assemblages at GR and SCR consisted mainly of largemouth bass, bluegill [*Lepomis macrochirus* (Rafinesque, 1810)], and black crappie [*Pomoxis nigromaculatus* (Lesueur, 1829)] (Fig. S7). In contrast, CR exhibited a more diverse assemblage, with 14 species observed. The fish assemblage at CR consisted of pelagic forage fish [e.g., inland silverside, *Menidia beryllina* (Cope, 1867); threadfin shad, *Dorosoma petenense* (Günther, 1867)], benthic feeders [e.g., brown bullhead, *Ameiurus nebulosus* (Lesueur, 1819); Sacramento sucker, *Catostomus occidentalis* (Ayres, 1854)], and carnivorous gamefish (largemouth bass). Like GR and SCR, AR consisted primarily of bluegill, largemouth bass, and black crappie, but also contained a notable population of threadfin shad. Almaden Reservoir’s fish assemblage represents a “midpoint” between the low-diversity assemblages of GR and SCR, and the highly diverse assemblage of CR.

### Suspended particulate matter

#### Stable isotopes in SPM

**Fig. 4:**
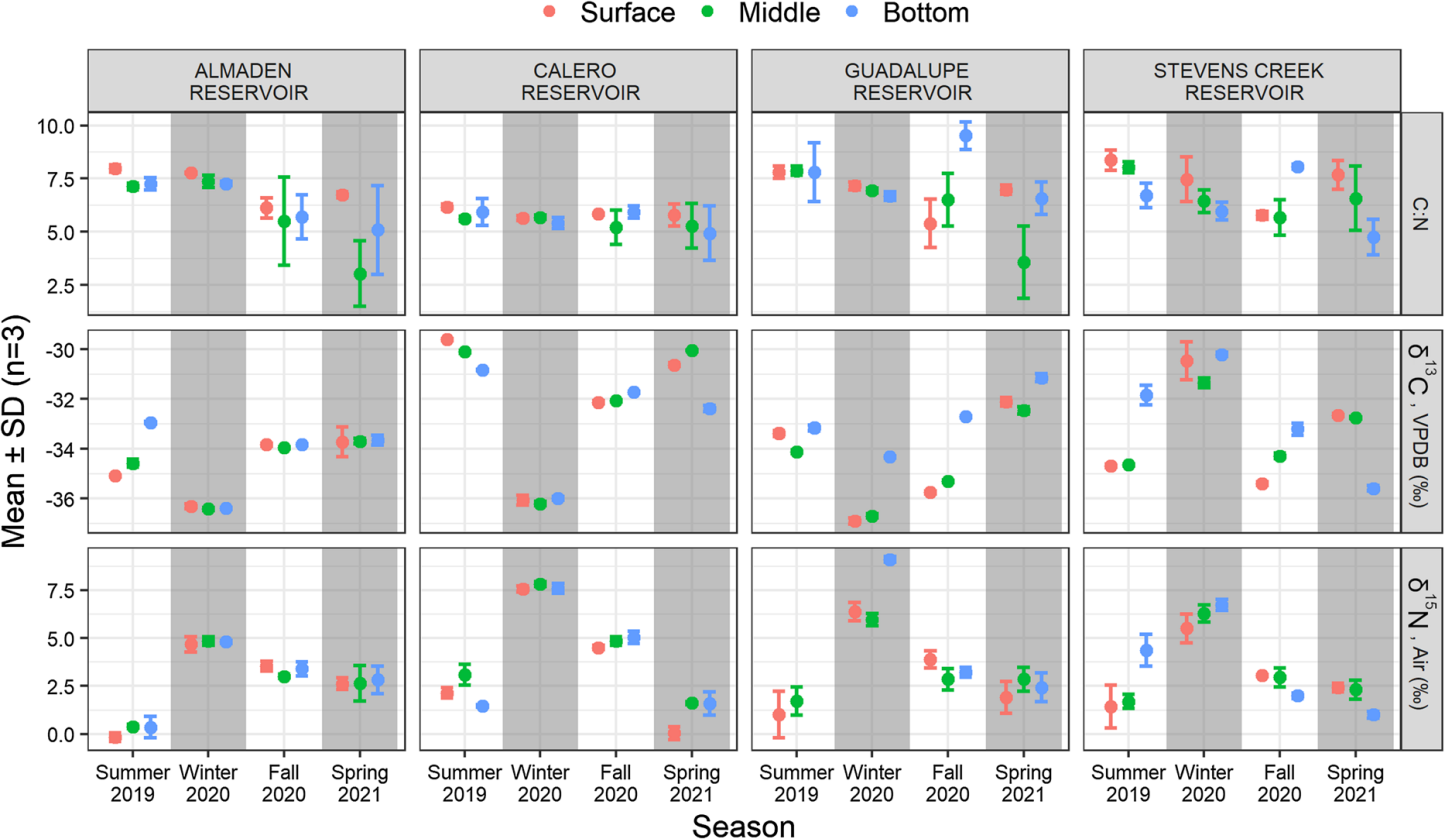
C:N ratios (top), δ^13^C (middle), and δ^15^N (bottom) measured in suspended particulate matter collected at three depths (colors) in each reservoir. δ^13^C values declined in winter while δ^15^N values peaked. Error bars show standard deviation of triplicate samples (some error bars are too small to be legible)

Stable C and N isotopes in SPM varied with reservoir, season, and collection depth. The reservoirs of the Guadalupe River Watershed, (AR, CR, GR) had similar patterns of δ^13^C values (Fig. 4). In each reservoir, SPM δ^13^C values peaked during the summer and spring sampling events but dropped to ~ −36‰ in winter 2020. Though CR exhibited a similar seasonal pattern as AR and GR, SPM in CR was generally more enriched in ^13^C. In SCR, SPM δ^13^C values displayed a different pattern, with the highest values (−31.5‰) measured in winter 2020. In some samples collected during thermal stratification, SPM δ^13^C values increased with sample depth, but the pattern was not consistent across reservoirs. Seasonal patterns in δ^15^N values were consistent among all four reservoirs, with peaks measured in winter 2020 and lower values in the spring and summer sampling events (Fig. 4). Winter 2020 peaks in SPM δ^15^N ranged from ~ 5‰ in AR to ~ 7.5‰ in CR, while summer lows ranged from ~ 0‰ in AR to ~ 2.5‰ in CR. Like δ^13^C values, there were sometimes a vertical gradient in SPM δ^15^N values, but the pattern was inconsistent across reservoirs. Carbon to N ratios, a measurement of food quality, were roughly constant in CR but varied seasonally in the other reservoirs (Fig. 4). In summer 2019, C:N ratios were generally highest, at ~ 6.25 in CR and ~ 8 in the other reservoirs. In the spring and fall sampling events, C:N ratios of SPM were more variable but generally lower.

#### Hg and MeHg in SPM

**Fig. 5:**
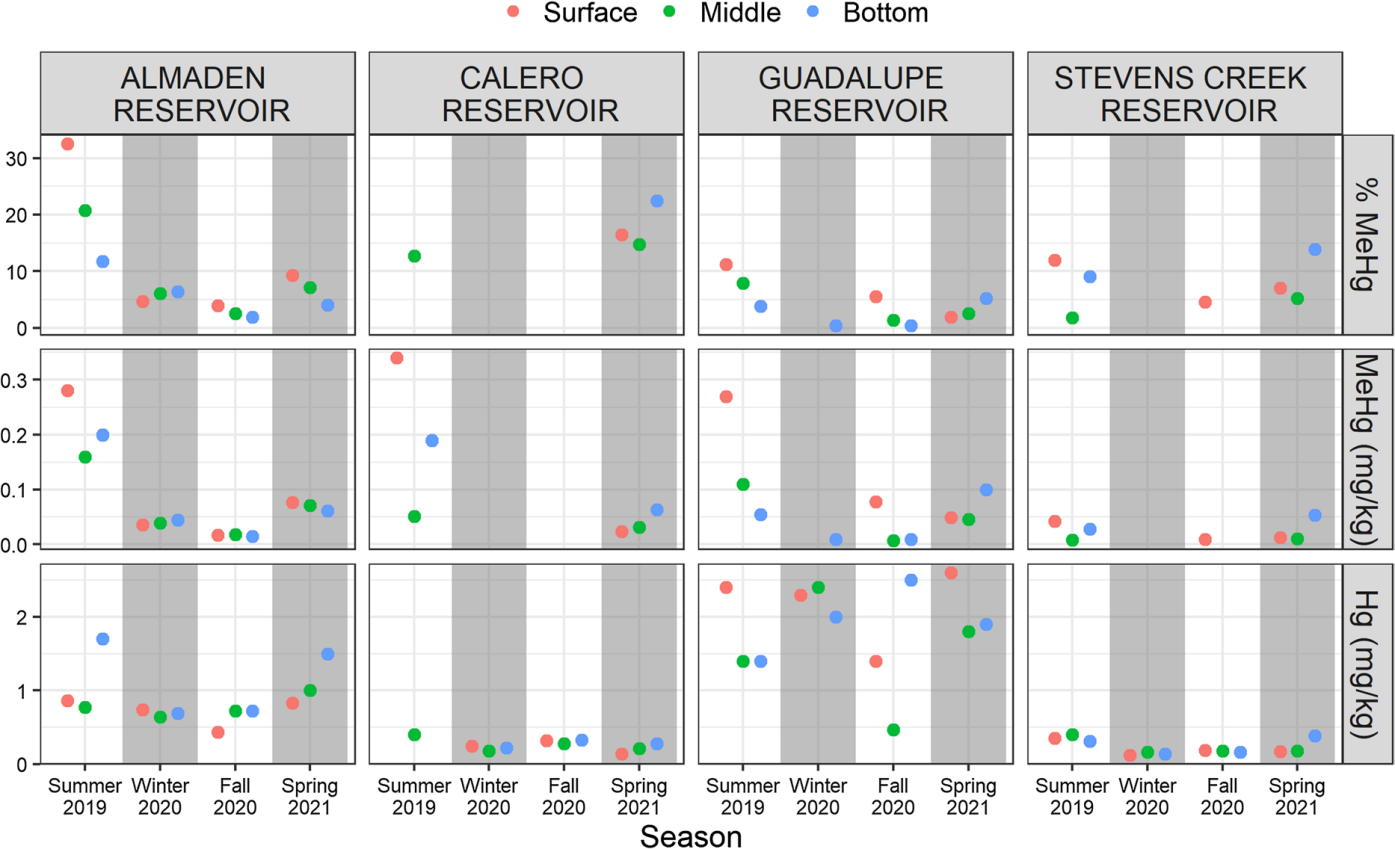
%MeHg (top), MeHg (middle), and Total Hg (bottom) measured in suspended particulate matter collected at three depths (colors) in each reservoir. MeHg concentrations peaked in summer and spring. Points represent single measurements. Values below detection limits are not shown. All concentrations are reported as dry weight. Percent MeHg (%MeHg) is calculated as [MeHg]/[total Hg]*100%

Mercury and MeHg were five to six orders of magnitude more concentrated in SPM than in reservoir water (Fig. 5). Nonetheless, MeHg was not detectable in some SPM samples collected in winter and fall 2020 from CR and SCR. Total Hg in SPM was < 0.5 mg/kg year-round in CR and SCR and did not vary notably with depth. In contrast, SPM total Hg in mine-impacted AR and GR was higher and more variable. In AR, total Hg in SPM was around 0.75 mg/kg, peaking to around 1.5 mg/kg in the deepest samples in summer 2019 and spring 2021. In GR, total Hg in SPM ranged from about 0.5 to 2.5 mg/kg, with high variability by depth. Patterns in MeHg in SPM were more consistent. Methylmercury in SPM peaked in summer 2019, and to a lesser extent in spring 2021 in each reservoir. Surprisingly, MeHg concentrations were consistently highest in the surface SPM samples in summer 2019 in all four reservoirs. Peak SPM MeHg concentrations in summer 2019 were similar in AR, CR, and GR, but notably lower in SCR. Percent MeHg in SPM was generally highest in summer and spring, often exceeding 5%. Percent MeHg in SPM was negatively correlated with SPM δ^15^N (Fig. S8).

### Zooplankton

#### Stable isotopes in zooplankton

**Fig. 6:**
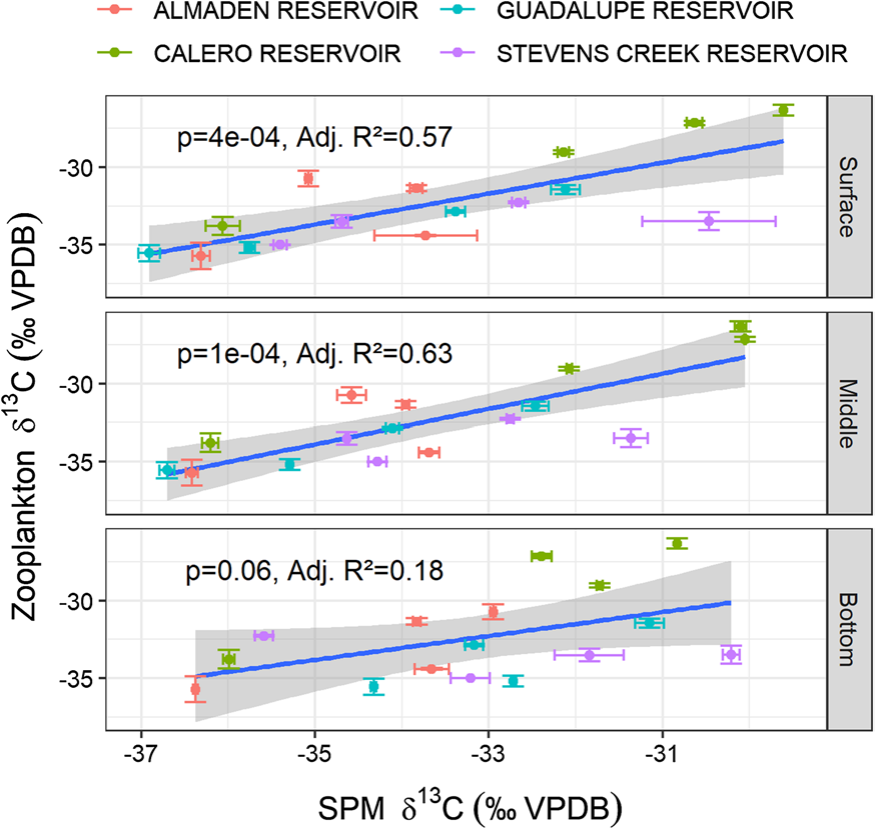
Linear correlations between δ^13^C measured in zooplankton composites and SPM collected from the surface, middle, and bottom sampling. Statistically significant relationships between δ^13^C in zooplankton and SPM collected from the surface and middle depths suggest that zooplankton feed in upper waters

Stable C and N isotope values of zooplankton composite samples varied by reservoir and collection season. Zooplankton δ^13^C was elevated in CR relative to the other reservoirs except in winter (Fig. S9). Zooplankton δ^13^C values were positively correlated with SPM δ^13^C values collected near the surface and middle of the reservoirs, but not with SPM collected lower in the water column (Fig. 6). Zooplankton samples were enriched in ^13^C with δ^13^C values 1.8 ± 1.4‰ (mean ± SD) above surface SPM samples. Zooplankton δ^15^N values appeared to have a positive association with SPM δ^15^N values in AR and CR, but not in GR or SCR (Fig. S10). In AR and CR, zooplankton were enriched in ^15^ N with δ^15^N values of 4.9 ± 2.2‰ above surface SPM samples. There was a significant correlation between the mass percent of copepods and δ^15^N values in the zooplankton composite samples (Fig. S10). Zooplankton C:N ratios were lower on average than C:N in SPM surface samples (4.7 ± 0.7 vs. 6.8 ± 1; mean ± SD).

#### Hg and MeHg in zooplankton

**Fig. 7:**
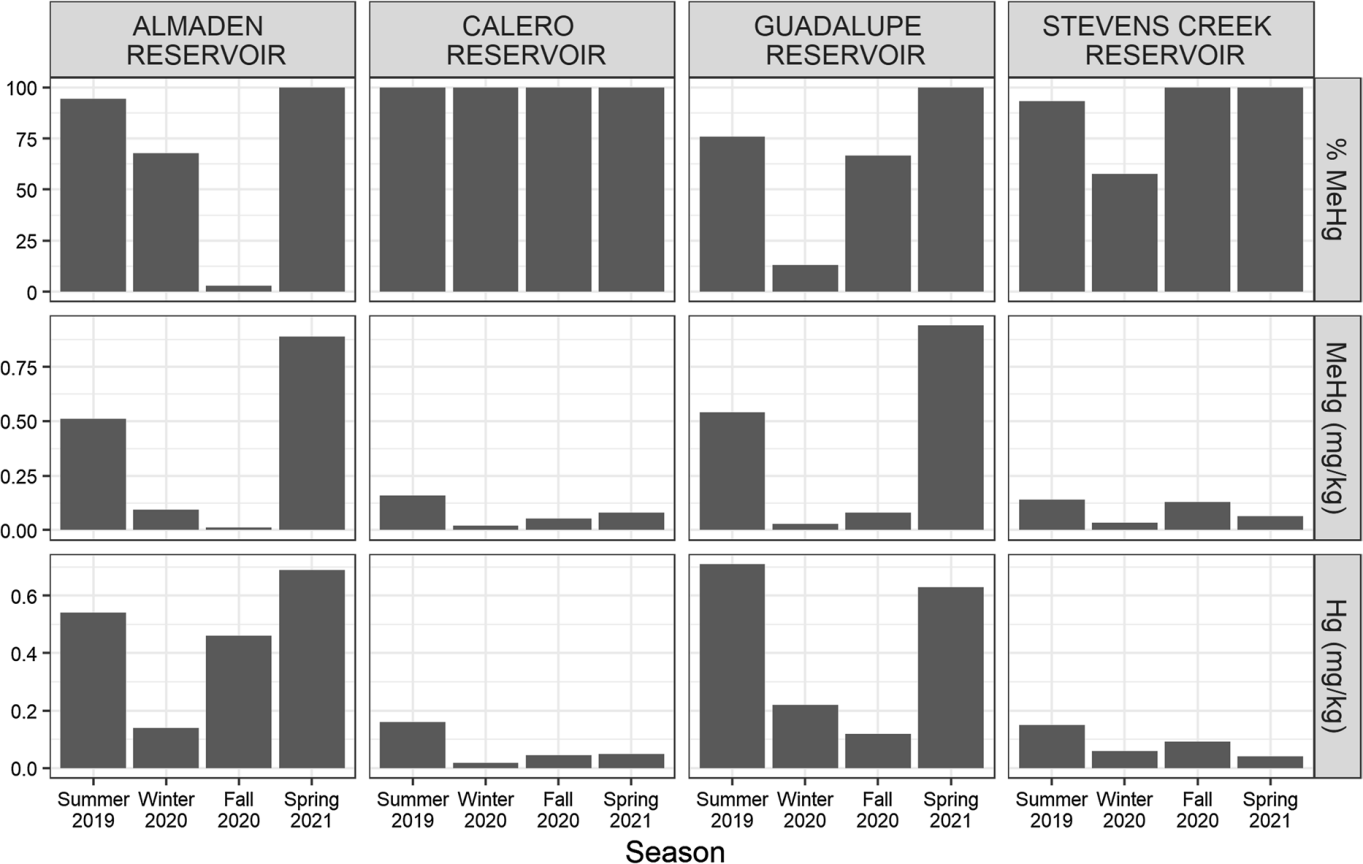
%MeHg (top), MeHg (middle), and Total Hg (bottom) measured in zooplankton composites collected in each reservoir. Zooplankton MeHg generally peaked in the summer and spring. Bar heights represent single measurements. All concentrations are reported in dry weight

Total Hg and MeHg in zooplankton varied by reservoir and by season. Total Hg concentrations in zooplankton were generally lower than total Hg in SPM, by 5 times on average. However, MeHg was generally more concentrated in zooplankton than in SPM, by 6 times on average. Differences between zooplankton and SPM MeHg were highly variable, resulting in no significant correlation between the two. The three reservoirs of the Guadalupe River Watershed (AR, CR, GR) had peak MeHg concentrations in zooplankton during summer 2019 and spring 2021 (Fig. 7). Peak MeHg in zooplankton were very similar in AR and GR, around 0.5 mg/kg in summer 2019 and 0.9 mg/kg in spring 2021. Zooplankton MeHg peaked in SCR in summer 2019 and fall 2019, both at concentrations around 0.13 mg/kg. In CR, 100% of the Hg in zooplankton was MeHg during all sampling events. However, %MeHg in zooplankton varied from around 2–100% in the other reservoirs dropping in winter 2020 in GR and SCR, and in fall 2020 in AR.

## Discussion

### Key differences between reservoir water chemistry and food webs

Water quality in the four reservoirs had key similarities and differences that are relevant to Hg cycling and bioaccumulation (Fig. 1d). Unsurprisingly, Hg and MeHg concentrations in water of mine-impacted AR and GR were considerably higher than in CR and SCR. However, CR and SCR contained much (2–3 times) higher concentrations of sulfate, which is known to stimulate MeHg production in aquatic sediments (Gilmour et al., 1992; Jeremiason, 2006). Additionally, eutrophic conditions observed in CR indicated by high phytoplankton density can promote high rates of MeHg production (Gray and Hines, 2009; Bravo et al., 2017). Thus, though total Hg concentrations are lower in CR and SCR, they may have favorable conditions for MeHg production and release into the water column. However, because MeHg production is thought to be first order with respect to the concentration of Hg, it is difficult to disentangle the effects of sulfate and trophic status vs. Hg concentration (Helmrich et al., 2021).

Phytoplankton species distribution and biomass density are key constraints on MeHg availability to pelagic ecosystems because they affect the uptake and concentration of MeHg at the base of the food web (Pickhardt et al., 2002; Lee and Fisher, 2016). Additionally, phytoplankton population can affect zooplankton growth and abundance, as cyanobacteria are thought to represent a low-quality and potentially toxic food source for grazers (Wilson et al., 2006; Martin-Cruezburg et al., 2009). Phytoplankton assemblages in the reservoirs varied seasonally but can be roughly separated into three archetypes: lower diversity-higher abundance (CR), lower diversity-lower abundance (GR and SCR), and higher diversity-lower abundance (AR) (Fig. 1a). Lower-diversity reservoirs consisted primarily of common cyanobacteria genera (e.g., *Dolichospermum*, *Aphanizomenon*, *Microcystis*) year-round, with minor populations of other phytoplankton groups (e.g., green algae, dinoflagellates, diatoms). In GR and SCR, these other groups increased in proportion seasonally, while cyanobacteria predominated throughout in CR. Cyanobacteria abundance and total algal density appeared to increase with residence time of water in the reservoir, likely due to nutrient accumulation and warmer, stagnant conditions that favor cyanobacteria growth (Romo et al., 2013). While cyanobacteria can facilitate the stabilization of Hg(II) in aquatic systems, high concentrations of cyanobacteria can also be associated with enhanced MeHg production and uptake (Lefebvre et al., 2007; Lázaro et al., 2013; Lee and Fisher, 2016). Additionally, high concentrations of cyanobacteria could decrease the somatic growth dilution of MeHg in zooplankton if the phytoplankton supply was insufficient (Karimi et al., 2007). Thus, these cyanobacteria-dominant reservoirs may present elevated risk for MeHg production and bioaccumulation. In contrast, AR contained populations of green algae, golden algae, and diatoms that comprised about 25–100% of the phytoplankton assemblage in each sampling event. Phytoplankton species often have lower surface area to volume ratios than common cyanobacteria, which could decrease passive MeHg uptake (Lee and Fisher, 2016). Green algae and diatom-dominant phytoplankton assemblages also constitute a higher quality food source for upper-level organisms, potentially increasing somatic growth dilution of MeHg in zooplankton (Wilson et al., 2006; Karimi et al., 2007; Martin-Cruezburg et al., 2009).

Zooplankton serve as a key trophic linkage between primary producers and planktivorous fish, representing an important control on MeHg bioaccumulation in the pelagic food web (Fisher and Reinfelder, 1995). Zooplankton abundance and community composition can influence their uptake and concentration of MeHg, ultimately affecting MeHg accumulation in fish (Pickhardt et al., 2005; Stewart et al., 2008). Zooplankton biomass density was highest at CR and SCR, with the greatest contribution from copepods due to their size and abundance. Like phytoplankton, zooplankton density and composition varied seasonally, but assemblages can be separated into two architypes: large-body dominant (AR, CR) and small-body dominant (GR, SCR) (Fig. 1b). Zooplankton assemblages in the large-body dominant reservoirs consisted primarily of large copepods of the order Cyclopoida (Burmeister, 1834) and their naupli. In contrast, GR and SCR had higher percentages of rotifers and smaller copepod genera such as *Microcyclops* (Claus, 1893). Both archetypes contained minor (10–30%) fractions of cladocera species, primarily of the genera *Bosmina* (Baird, 1845) and *Daphnia* (Müller, 1785). Rotifer abundance decreased with increasing phytoplankton density. This result disagrees with other studies that showed increased rotifer density resulting from eutrophication (Blancher, 1984; Ejsmont-Karabin, 2012). Because rotifers can have lower MeHg concentrations than zooplankton species of higher trophic level, increases in algal biomass could indirectly increase MeHg concentrations in the zooplankton community (Stewart et al., 2008). Furthermore, increases in abundance of carnivorous copepod species under high algae density could add an additional trophic step in the food web and increase MeHg concentrations in zooplankton (Cabana et al., 1994).

Fish abundance and species distribution are important factors influencing plankton populations and MeHg bioaccumulation. The fish assemblages of GR and SCR primarily consisted of opportunistic and piscivorous feeders (e.g., bluegill, largemouth bass) that had no clear linkage to planktonic food web. In contrast, AR and CR contained planktivorous forage fish (e.g., threadfin shad, inland silverside) (Fig. 1c). Unlike GR and SCR, which had small-dominant zooplankton assemblages, AR and CR contained higher abundances of large copepods and cladocera, which may explain the presence of planktivorous fish. In addition to connecting the planktonic food web to predatory fish, planktivorous fish can provide a top-down control on the plankton assemblage and density in lakes (Li et al., 2020). Predation by fish could explain why zooplankton abundance is relatively low in AR and CR despite high phytoplankton diversity in AR and abundance in CR.

### Patterns in plankton and suspended particulate matter

Plankton density and community structure varied seasonally in each reservoir. There was no consistent pattern in phytoplankton productivity among the reservoirs, with peaks in phytoplankton biomass identified in summer (AR, CR), winter (GR), fall (GR, SCR), and spring (CR) (Fig. 2). Likewise, there did not appear to be a consistent seasonal pattern in phytoplankton assemblages. Surprisingly, none of the water quality variables measured were correlated with phytoplankton biomass. It is likely that nutrient and/or light limitation were key factors controlling bloom events (Han et al., 2021). Patterns in zooplankton density were consistent in CR, GR, and SCR, with peaks measured during the summer and fall sampling events (Fig. 3). However, we found no correlation between biomass densities of phytoplankton or cyanobacteria and zooplankton, suggesting that the zooplankton population is not limited by food availability. Predation by planktivorous fish and carnivorous zooplankton is likely an important top-down control on zooplankton density that obscures the relationship between the phytoplankton and zooplankton communities (Sinstro, 2010; Li et al., 2020).

Suspended particulate matter samples were depleted in ^13^C relative to known isotope values reported in phytoplankton (δ^13^C from −18 to −25 ‰) (Popp et al., 1998; Hayes, 2001). However, SPM δ^13^C values fell within the range of literature values of lake SPM (Cattaneo et al., 2004; Taipale et al., 2016; Lammers et al., 2017). This difference in δ^13^C values likely reflects contributions from allochthonous organic matter, which is expected to be relatively depleted in ^13^C (Wang and Druffel, 2001; Hedges et al., 1997). The large decrease in SPM δ^13^C values observed in winter 2020 likely reflects allochthonous loading of organic during the wet season, which has lower δ^13^C values associated with terrestrial vegetation (Fig. 5). Conversely, δ^15^N values in SPM was highest in winter 2020 and lower in other seasons. Differences in SPM δ^15^N values can reflect the species of dissolved N present (NO_3_^−^: 3–7‰, NH_4_^+^: 6–8‰, and atmospheric N_2_: 0‰ per Miyake and Wada, 1967) or the nitrogen source (Kendall et al., 2001). Relatively low SPM δ^15^N values in summer and spring likely reflects the high abundance of cyanobacteria species that fix atmospheric nitrogen (Minigawa & Wada, 1984). High (> 5‰) δ^15^N values in SPM measured in winter 2020 suggests external loading of particulate organic nitrogen, and/or phytoplankton reliance on dissolved N species.

Stable C and N isotope values in zooplankton reflected their food source and community structure. Zooplankton δ^13^C values were significantly associated with SPM δ^13^C values collected from the surface and mid-water column (Fig. 8). This correspondence indicates that zooplankton primarily feed in the pelagic zone. However, there appeared to be no association between zooplankton δ^15^N and SPM δ^15^N values in GR and SCR. Instead, zooplankton δ^15^N values were related to the zooplankton community structure, with δ^15^N values increasing with the mass percentage of copepods present. Carnivory of other zooplankton species by copepods could increase the trophic fractionation of N disproportionally to C, causing a misalignment of δ^15^N and δ^13^C values (Post, 2002). It is likely that variations in zooplankton δ^15^N values in GR and SCR reflect seasonal variability in N source and carnivory by zooplankton. Zooplankton carnivory effectively adds a trophic step to the pelagic food web, which could increase MeHg bioaccumulation (Cabana et al., 1994).

### Patterns in Hg bioaccumulation

Suspended particulate matter accumulated Hg and MeHg at much higher concentrations (~ 1 million times) than were present in reservoir water. However, we did not observe predictive relationships between Hg or MeHg measured in SPM and water at the time of collection. This was especially true when considering FP MeHg as representative of the pool of MeHg in water because FP MeHg was highly variable and often not detectable. The concentration of Hg species in SPM represents a time-averaged snapshot of Hg exposure influenced by factors such as Hg concentration and speciation in water, SPM origin and age, and phytoplankton density and composition. Each of these factors can vary on timescales from days to months. Thus, a single water measurement of Hg or MeHg taken during the collection of SPM will not represent the Hg time history associated with the SPM sample and cannot accurately explain fine-timescale changes in Hg uptake by SPM. Increasing sampling frequency to monthly or less could help constrain water-SPM biomagnification.

Consistent total Hg concentrations in SPM (~ 0.25 mg/kg) measured year-round in CR and SCR suggests that a relatively unchanging input (e.g., atmospheric deposition) dominates Hg loading to these reservoirs (Fig. 5). In contrast, the higher (0.5–2.5 mg/kg) and more variable Hg concentrations measured in SPM collected from AR and GR suggests that Hg loading varies with inflow and reservoir conditions. Methylmercury concentrations in SPM peaked during the summer and spring sampling events in each reservoir. This is consistent with studies that have noted enhanced MeHg production in stratified reservoirs during the spring and early summer prior to the establishment of highly reducing conditions (Beutel et al., 2020; Fuhrmann et al., 2021). We did not measure elevated Hg or MeHg in biota following fall turnover, as has been noted in other studies (Slotton et al., 1995; Herrin et al., 1998). It is likely that bottom discharge from the reservoirs and hypolimnetic oxygenation kept MeHg concentrations relatively low in bottom waters, such that MeHg bioaccumulation was more dependent on MeHg production occurring in the littoral zone and/or water column than on seasonal inputs from the hypolimnion during reservoir mixing (McCord et al., 2016; Seelos et al., 2021). This hypothesis is supported by higher MeHg concentrations measured in surface SPM samples in summer 2019 compared to those measured closer to the bottom. Percent MeHg in SPM was negatively associated with δ^15^N values in SPM, likely due to enhanced MeHg production at times when SPM was autochthonous in origin, or preferential MeHg uptake relative to inorganic Hg (Fig. S8). Phytoplankton-derived dissolved organic matter is known to support relatively high rates of Hg methylation (Bravo et al., 2017).

Zooplankton assimilated MeHg much more efficiently than inorganic Hg. Whereas total Hg concentrations in zooplankton were about 5 times lower than in SPM, MeHg concentrations averaged 5–6 × times higher (Fig. 7). This result agrees with other studies showing high rates of MeHg assimilation by zooplankton compared to inorganic Hg (Lee and Fisher, 2016; Gosnell et al., 2021). The fraction of total Hg in zooplankton as MeHg was typically > 50% with lower fractions generally occurring during the winter when MeHg production was low. Despite similar concentrations of MeHg in surface SPM of AR, CR, and GR during summer 2019, zooplankton MeHg concentrations were much lower in CR. Zooplankton may have selectively fed in the mid-water column where phytoplankton density was ~ 50% lower and MeHg concentrations were ~ 0.05 mg/kg. Surprisingly, zooplankton in AR and GR contained the highest MeHg concentrations during spring 2021 when MeHg in SPM was relatively low. This result corresponded with decreased zooplankton density and lower C:N in zooplankton composites (Fig. S9). It is possible that low zooplankton densities decreased grazer competition and allowed for more selective feeding on “fresh” phytoplankton that likely contained higher concentrations of MeHg. Conversely, high zooplankton densities measured in summer 2019 could have led to lower selectivity by zooplankton, including feeding on older detritus that may have had lower MeHg concentrations compared to fresh algal biomass. Zooplankton feed selectively on the best available resources, perhaps making bulk SPM and phytoplankton counts poor proxies for zooplankton diet (Sailley et al., 2015; Meunier et al., 2016). This was evident in the poor correlations between MeHg or δ^15^N in SPM and zooplankton composites.

## Conclusion

Building understanding of the factors that contribute to MeHg bioaccumulation in reservoirs is crucial to developing management strategies aimed at lowering Hg bioaccumulation, particularly because MeHg concentrations in surface waters are often poorly correlated with MeHg concentrations in biota. This study presents one approach for assessing the drivers of MeHg bioaccumulation in reservoir systems using a diverse set of chemical and ecological data. Nonmetric multidimensional scaling analysis revealed key similarities and differences between four monomictic reservoirs that could affect MeHg production and bioaccumulation. Mine-impacted AR and GR had high Hg and MeHg concentrations, but likely less favorable conditions for MeHg production than CR and SCR, which had higher sulfate concentrations and phytoplankton productivity. Significant overlaps in the plankton and fish assemblages of GR and SCR suggest ecological similarities that could affect Hg bioaccumulation in these reservoirs. Almaden and Calero reservoirs likewise had similar plankton and fish assemblages, with the notable presence of pelagic forage fish that could efficiently connect the planktonic food web with upper trophic level fish. Stable isotope results indicated that zooplankton likely fed primarily in the upper water column of the reservoirs, but zooplankton biomass did not seem to be limited by food availability. Instead, fish and carnivorous zooplankton may present a top-down control on the zooplankton population. Percent MeHg in SPM was negatively associated with δ^15^N values, suggesting that “fresh” algal biomass could support preferential MeHg uptake (relative to total Hg) or enhanced MeHg production. Methylmercury accumulated in SPM during the spring and summer seasons in each reservoir, when conditions were most favorable to MeHg production. Surprisingly, though MeHg concentrations in SPM were highest during the summer, zooplankton MeHg was highest in the spring in AR and GR. This may have been due to low zooplankton density, which could decrease feeding competition and allow for more ingestion of MeHg per organism. Methylmercury and stable isotope data in biota suggest that bulk SPM may not represent dietary inputs to zooplankton, which feed selectively and adjust their diet based on the best available food source. Overall, our results demonstrate the seasonal patterns in MeHg introduction into the pelagic food web, and ecological similarities in AR and CR, and in GR and SCR.

While the combination of various, diverse data sources is a promising method for exploring MeHg bioaccumulation in aquatic systems, site specific variability, particularly in Hg and MeHg concentrations measured in biota, confounded efforts to identify general drivers across all study reservoirs. Intensive, high-temporal-resolution studies using replicate measurements of MeHg and Hg in biota, though costly, may be necessary to identify management opportunities at each site. To best use limited resources, ordination analysis of water quality and ecological characteristics may be used group sites into similar categories from which one representative site may be selected. One important but missing (on account of COVID-19) component of our study was the inclusion of fish and benthic macroinvertebrate sampling for MeHg and stable isotopes of C and N. The inclusion of fish and benthic macroinvertebrate sampling would yield a complete dataset of all potential tropic pathways for MeHg bioaccumulation in reservoirs and should be considered in future studies.

## Supplementary Information

Below is the link to the electronic supplementary material.

Supplementary file1 (PDF 2558 kb)

## Data Availability

The datasets generated during and/or analyzed during the current study are available from the corresponding author on reasonable request.
