# Supplementary material for: Plankton population dynamics and methylmercury bioaccumulation in the pelagic food web of mine-impacted surface water reservoirs
Source: Hydrobiologia. 2022 Oct 1;849(21):4803–22. doi: 10.1007/s10750-022-05018-0 (PMC9526464; doi:10.1007/s10750-022-05018-0)
Supplement: Supplementary file 1 — Supplementary file1 (PDF 2558 kb) [file 10750_2022_5018_MOESM1_ESM.pdf]

Supporting Information

Plankton Population Dynamics and Methylmercury Bioaccumulation in the Pelagic Food Web of Mine-Impacted Surface Water Reservoirs

Mark Seelos[1, 2*], Marc Beutel[1], Stephen McCord[3], Sora Kim[4], Katie Vigil[5]

[1] Environmental Systems Graduate Program, University of California Merced, Merced, CA, 95343, USA

[2] Valley Water, San Jose, CA, 95118, USA

[3] McCord Environmental, Inc., Davis, CA, 95616, USA

[4]Department of Life and Environmental Sciences, University of California Merced, Merced, CA, 95343, USA

[5]Tulane University School of Public Health and Tropical Medicine, Dept. of Global Environmental Health, New Orleans, LA, 70112, USA

*Corresponding Author: Mark Seelos
Email: mseelos@valleywater.org

Contents

Figure S1. Map of upper the upper Guadalupe River Watershed hydrologic system. 2

Table S1. Physical and biogeochemical characteristics of the study reservoirs.. 3

Figure S2. Oxygenation system operation, dissolved oxygen heat maps, and suspended particulate matter sample depths for each reservoir over the study period.................................. 4

Figure S3. Temperature heat maps and suspended particulate matter sample depths for each reservoir over the study period.. 5

Table S2. Biovolumes used for phytoplankton taxa to convert counts to biomass.. 6

Table S3. Per-organism dry weight values used to convert zooplankton counts to biomass. 7

Figure S4. Phytoplakton and zooplankton assemblages by functional classification.................... 8

Figure S5. Nonmetric Multidimensional Scaling (NMDS) analysis of phytoplankton (A) and zooplankton (B) with environmental variable vectors.. 9

Figure S6. Average fish catch per minute (all species) in each reservoir (2012 to present).. 10

Figure S7. Average fish catch per minute of individual fish species in each reservoir (2012 to present).. 11

Figure S8. δ^{15}N vs. % MeHg in SPM samples collected from surface waters.. 12

Figure S9. C:N ratios, $\delta^{13}C$, and $\delta^{15}N$ measured in zooplankton composites 13

Figure S10. $\delta^{15}N$ in zooplankton vs. $\delta^{15}N$ in SPM measured at the surface, middle, and bottom sample depths. 14

Figure S11. $\delta^{15}N$ in zooplankton composites vs. the mass percentage of copepods in the composite.. 15

Figure S1. Map of upper the upper Guadalupe River Watershed hydrologic system, including Almaden Reservoir (AR), Calero Reservoir (CR), Guadalupe Reservoir (GR), major streams, canals, and pipelines. Inset A shows the site location in the state of California. Inset B shows Stevens Creek Reservoir (SCR), located 12 kilometers northwest of GR.

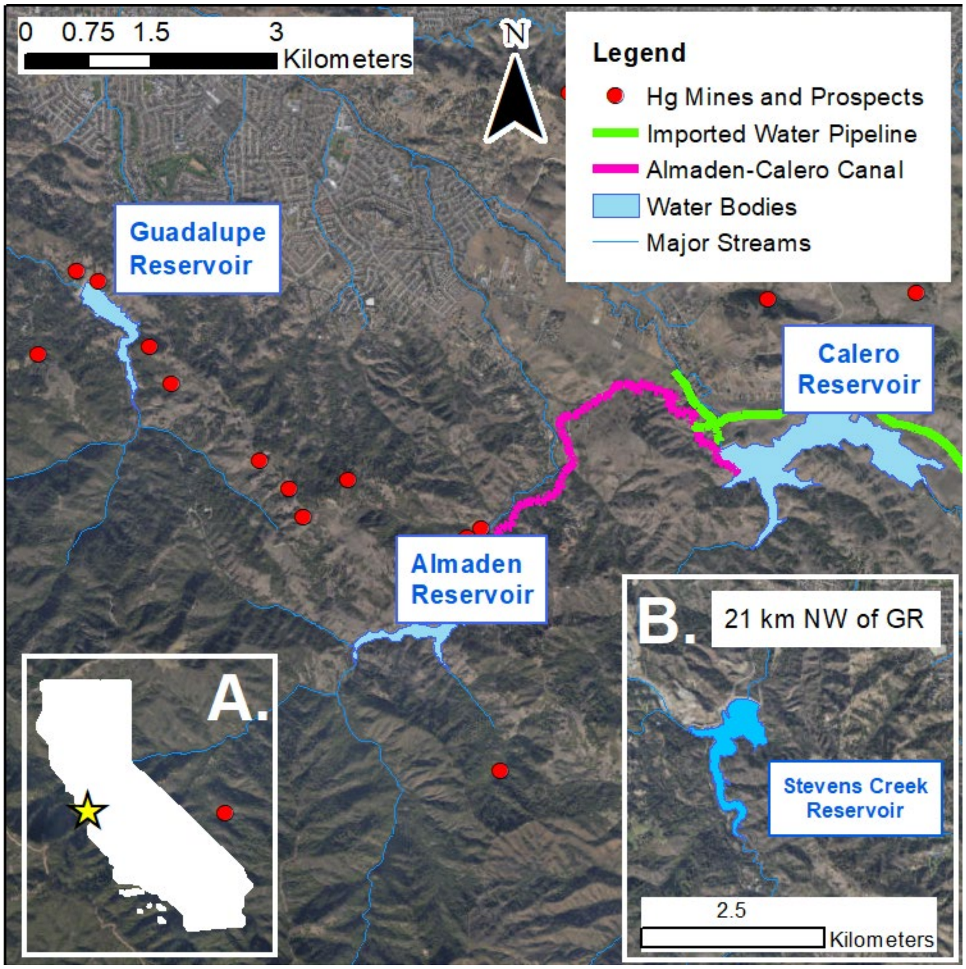

Table S1. Physical (top) and biogeochemical (bottom) characteristics of the study reservoirs. Measurements with error reported are mean ± standard deviation.

		Almaden (AR)	Calero (CR)	Guadalupe (GR)	Stevens Creek (SCR)
Physical	Water Source	Local	Imported, Minor Local	Local	Local
	Catchment Area (ha)	3102	1892	1543	4469
	Max Volume (m^3)	1.96E+06	1.23E+07	4.21E+06	3.87E+06
	Mean Depth (m)	9.8	10.4	18.3	11
	Max Depth (m)	20	25	30	20
	Residence Time (d)	138±3	497±16	240±5	216±4
Biogeochemical	Hg Source	Mine Runoff	AR, Geology, Atmospheric	Mine Runoff	Geology, Atmospheric
	Hg (ng/L, surface)	13.1±4.2	12.7±7.7	13.3±1.2	6.4±2.4
	Hg (ng/L, bottom)	11±1.1	6.2±1.1	31.3±1.3	8.1±1.3
	MeHg (ng/L, surface)	0.6±0.03	0.2±0.01	0.5±0.03	0.1±0.01
	MeHg (ng/L, bottom)	0.9±0.1	0.9±0.1	5.5±0.6	0.4±0.1
	Chl a (ug/L, surface)	5.1±0.4	10.3±0.4	3.1±0.2	4±0.3

Figure S2. Oxygenation system operation, dissolved oxygen heat maps, and suspended particulate matter (SPM) sample depths for each reservoir over the study period. Dissolved oxygen heat maps were made by linearly interpolating vertical profile measurements (dashed lines) collected at the deepest portions of the reservoirs. Data gaps due to COVID-19 pandemic.

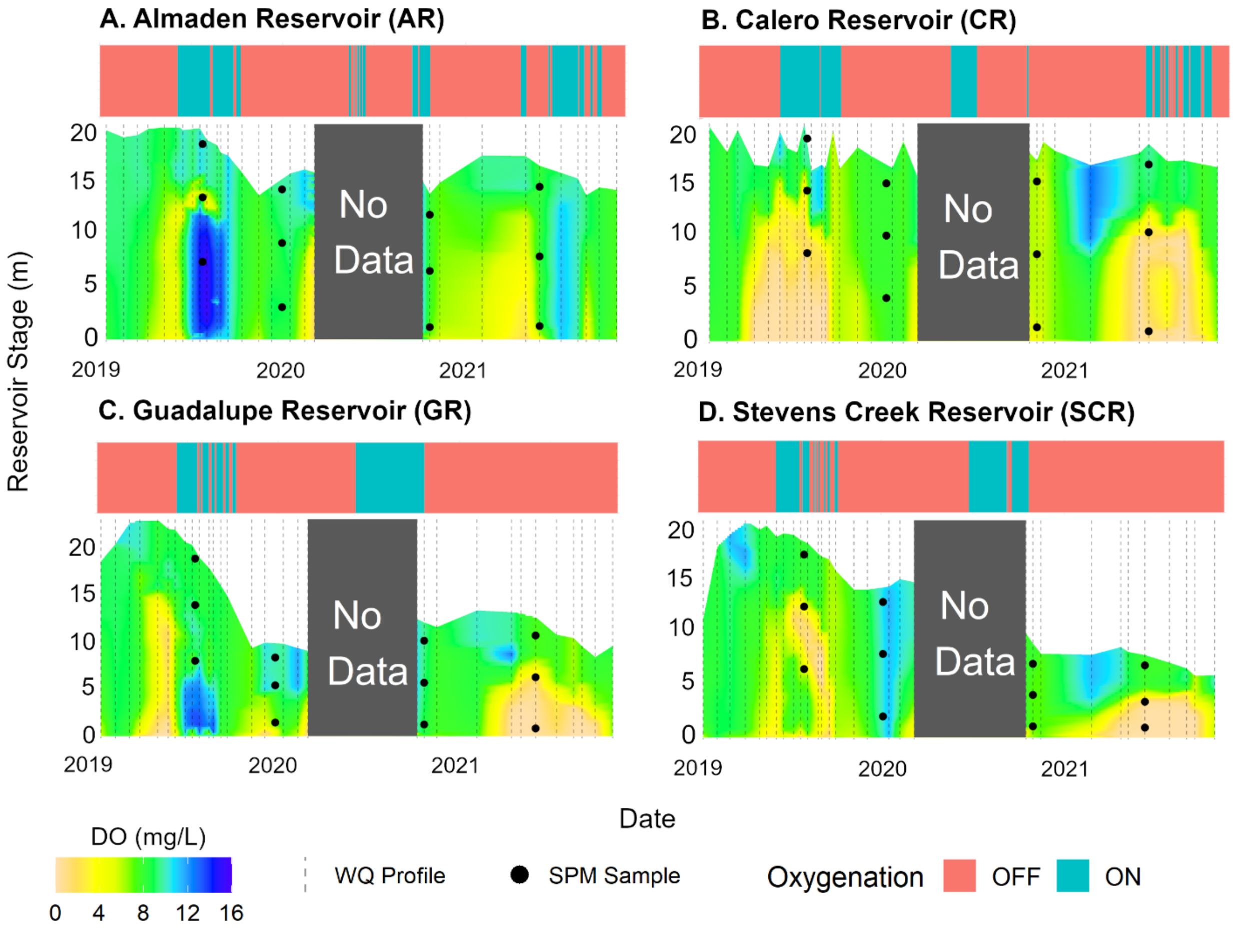

Figure S3. Temperature heat maps and suspended particulate matter (SPM) sample depths for each reservoir over the study period. Temperature heat maps were made by linearly interpolating vertical profile measurements (dashed lines) collected at the deepest portions of the reservoirs. Data gaps due to COVID-19 pandemic.

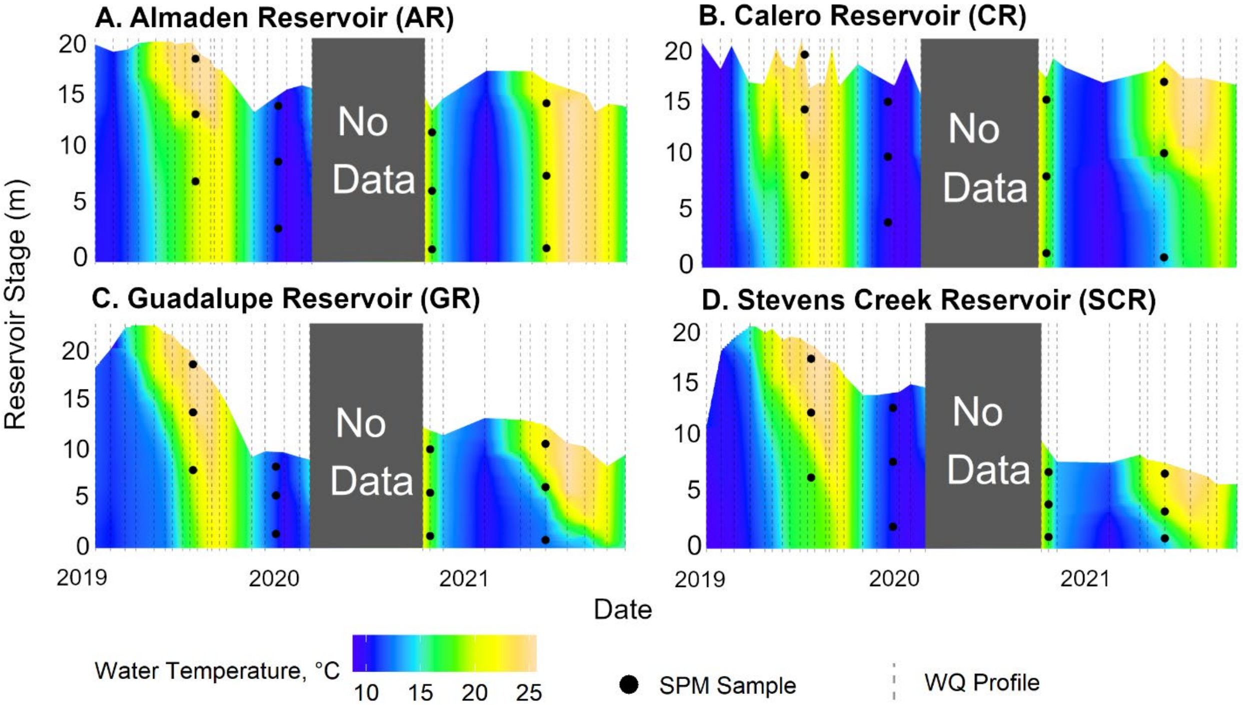

Table S2. Biovolumes used for phytoplankton taxa to convert counts to biomass. We assumed a density of 1g/cm^3.

Taxon	Classification	Volume (μm^3)	Calculated Mass (ng)	Volume Reference
Anabaena aphanizomenoides	cyanobacteria	3349	3.349	Olenina et al. 2006
Anabaenopsis	cyanobacteria	1060	1.06	Olenina et al. 2006
Aphanizomenon	cyanobacteria	707	0.707	Olenina et al. 2006
Aphanothece sp.	cyanobacteria	6	0.006	Olenina et al. 2006
Asterionella sp.	diatoms	858	0.858	Olenina et al. 2006
Ceratium sp.	dinoflagellates	28964	28.964	Olenina et al. 2006
Closterium sp.	green algae	5233	5.233	Olenina et al. 2006
Cryptomonad sp.	green algae	82	0.082	Olenina et al. 2006
Cyclotella	diatoms	1722	1.722	Olenina et al. 2006
Cylindrospermopsis	cyanobacteria	96	0.096	Hong et al. 2006
Cylindrospermopsis catemaco	cyanobacteria	96	0.096	Hong et al. 2006
Dinobryon sp.	golden algae	19	0.019	Olenina et al. 2006
Dolichospermum sp.	cyanobacteria	955	0.955	Matthews 2016
Eudorina sp.	green algae	310	0.31	Olenina et al. 2006
Fragilaria sp.	diatoms	688	0.688	Olenina et al. 2006
Melosira	diatoms	653	0.653	Olenina et al. 2006
Microcystis	cyanobacteria	4	0.004	Olenina et al. 2006
Nitzschia	diatoms	1600	1.6	Olenina et al. 2006
Oocystis	green algae	158	0.158	Olenina et al. 2006
Pandorina	green algae	697	0.697	Olenina et al. 2006
Planktothrix	cyanobacteria	217	0.217	Olenina et al. 2006
Pseudanabaena sp.	cyanobacteria	34	0.034	Olenina et al. 2006
Raphidiopsis	cyanobacteria	263	0.263	Jia et al. 2020
Scenedesmus	green algae	212	0.212	Olenina et al. 2006

Stephanodiscus sp.	diatoms	114	0.114	Olenina et al. 2006
Synedra sp.	diatoms	135	0.135	Olenina et al. 2006
Tetraedron sp.	green algae	224	0.224	Olenina et al. 2006
Woronichinia sp.	cyanobacteria	6	0.006	Olenina et al. 2006

Table S3. Per-organism dry weight values used to convert zooplankton counts to biomass.

**Taxa**	**Classification**	**Dry Weight (μg)**	**Source**
Asplanchna sp.	rotifera	0.2	Wetzel, 2001
Bosmina longirostris	cladocera	2	Wetzel, 2001
Bosmina sp.	cladocera	2	Wetzel, 2001
Ceriodaphnia sp.	cladocera	3	Wetzel, 2001
Chaoborus flavicans	diptera	0.002	Dumont and Balvay, 1979
Chaoborus sp.	diptera	0.002	Dumont and Balvay, 1979
Conochilus sp.	rotifera	0.08	Wetzel, 2001
Conochilus unicornis	rotifera	0.08	Wetzel, 2001
Cyclopoid naupli	copepods	15.5	Wetzel, 2001
Cyclops	copepods	15.5	Wetzel, 2001
Daphnia lumholtzi	cladocera	18.5	Wetzel, 2001
Daphnia sp.	cladocera	18.5	Wetzel, 2001
Diacyclops	copepods	15.5	Wetzel, 2001
Diacyclops thomasi	copepods	15.5	Wetzel, 2001
Eurytemora	copepods	15.5	estimated as Cyclops
Hydracarina sp.	arachnid	18.5	estimated as Daphnia
Keratella sp.	rotifera	0.075	Wetzel, 2001
Kellicottia sp.	rotifera	0.085	Wetzel, 2001
Limnocalanus	copepods	18.5	estimated as Daphnia
Limnocalanus macrurus	copepods	32.5	Apollonio and Sauros, 2012
Microcyclops rubellus	copepods	15.5	estimated as Cyclops
Microcyclops	copepods	15.5	estimated as Cyclops
Polyarthra sp.	rotifera	0.06	Wetzel, 2001
Skistodiaptomus sp.	copepods	15.5	estimated as Cyclops

Figure S4. Phytoplakton (A) and Zooplankton (B) assemblages by functional classification. Values are reported as a percentage of the total count of individual organisms.

A. Phytoplankton

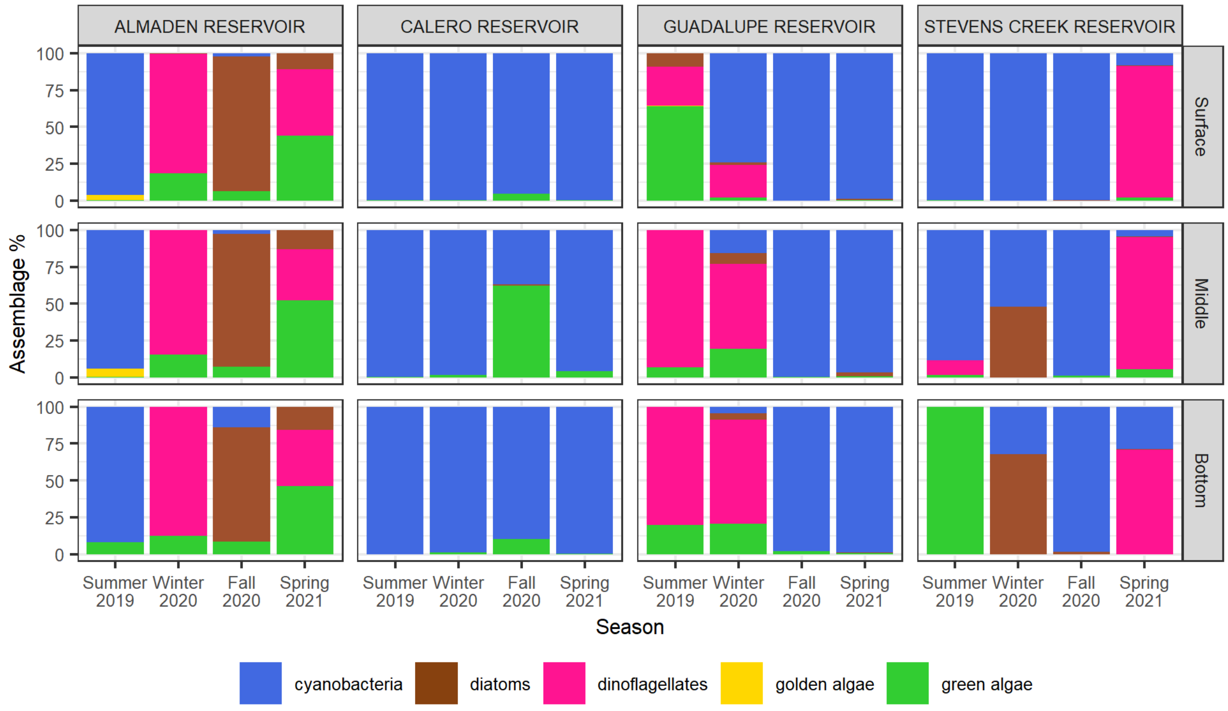

B. Zooplankton

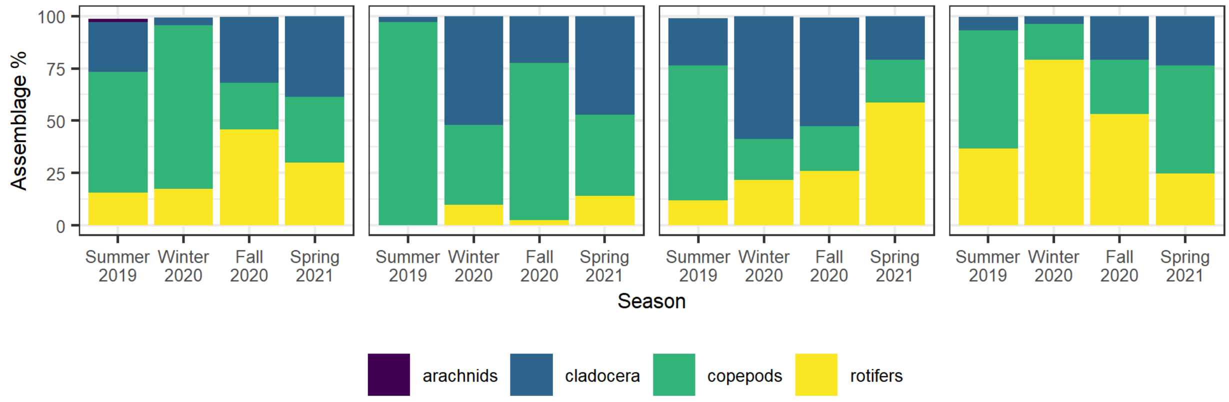

Figure S5. Nonmetric Multidimensional Scaling (NMDS) analysis of phytoplankton (A) and zooplankton (B). Each point is an individual assemblage of organisms. The color of the point represents the reservoir. The shape of the point represents the collection season. The size of the point (A only) represents the sample depth. Functional classes of organisms are shown as red text, with assemblage points closer to the text containing a greater proportion of organisms in that functional class. Explanatory variables that were significantly ($p<0.05$) associated with taxa distributions are shown as black vectors, with the direction of the vector aligned with the gradient of variation. The number of reduced dimensions (k) and Kruskal's Stress (stress) are shown in the bottom-right of each plot.

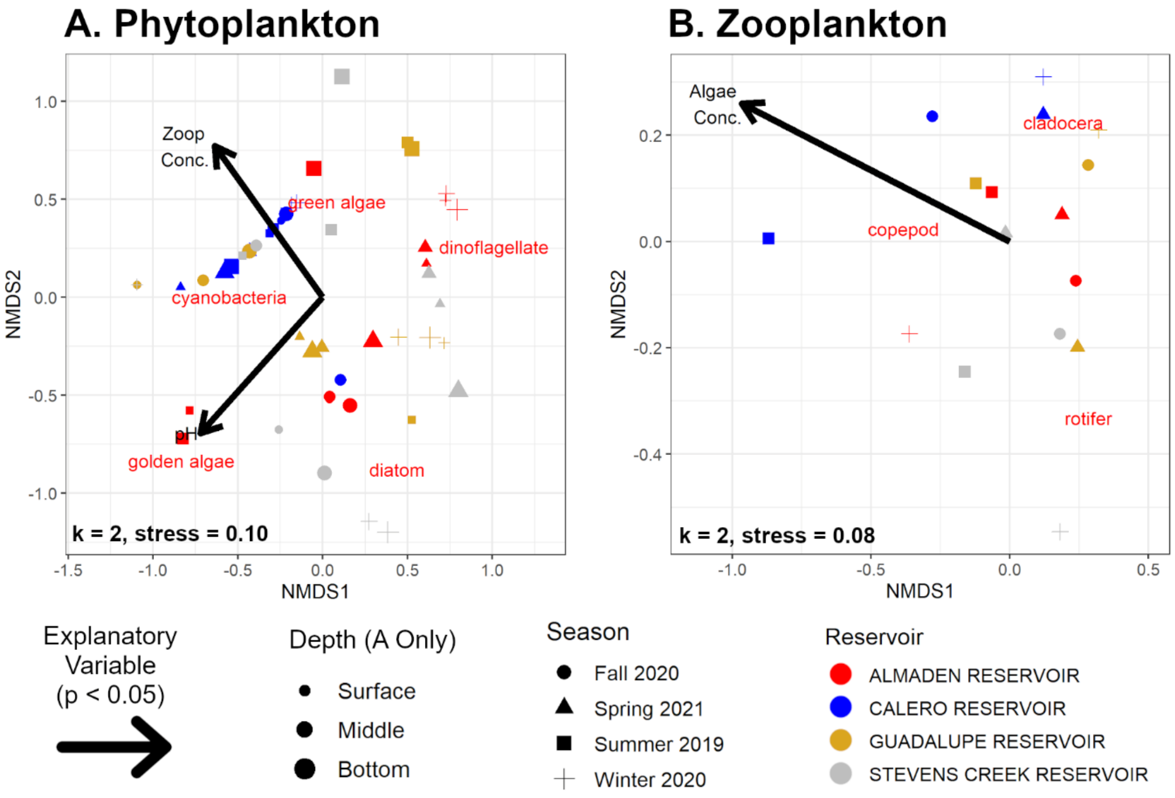

Figure S6. Average fish catch per minute (all species) in each reservoir (2012 to present). One observation corresponds to one sampling event. To yield comparable results, only samples collected using boat electrofishing with four netters are shown. Red letters indicate which reservoirs have statistically different ($p<0.05$, Kruskal Wallis test) catch per minute.

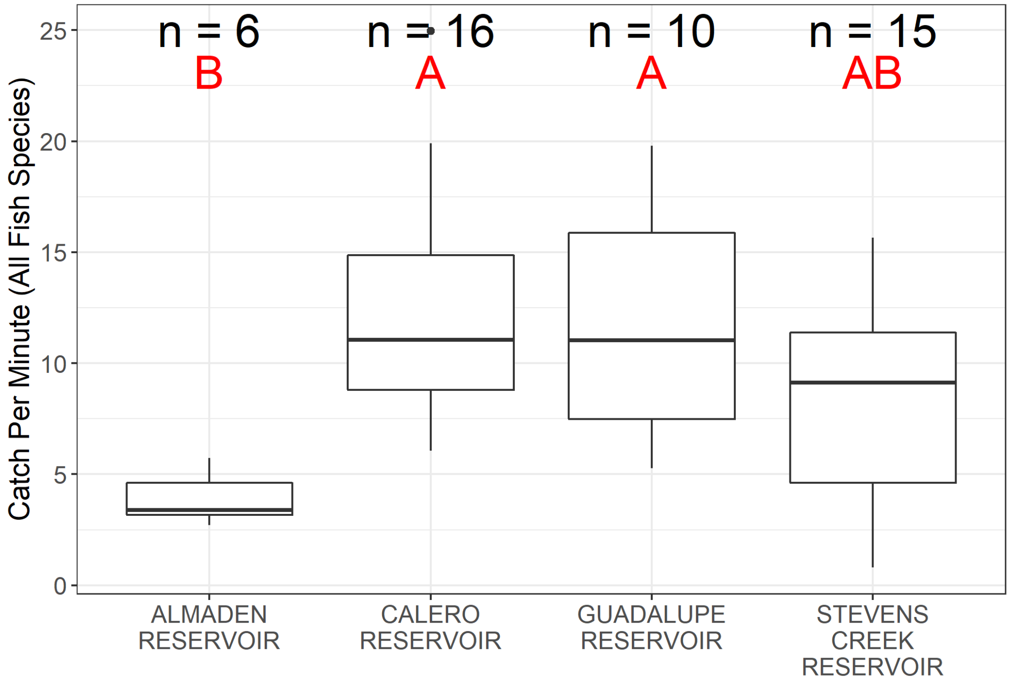

Figure S7. Average fish catch per minute of individual fish species in each reservoir (2012 to present). One observation corresponds to one sampling event. To yield comparable results, only samples collected using boat electrofishing with four netters are shown. The bar height is the mean and the error bars show the mean + 1 standard deviation.

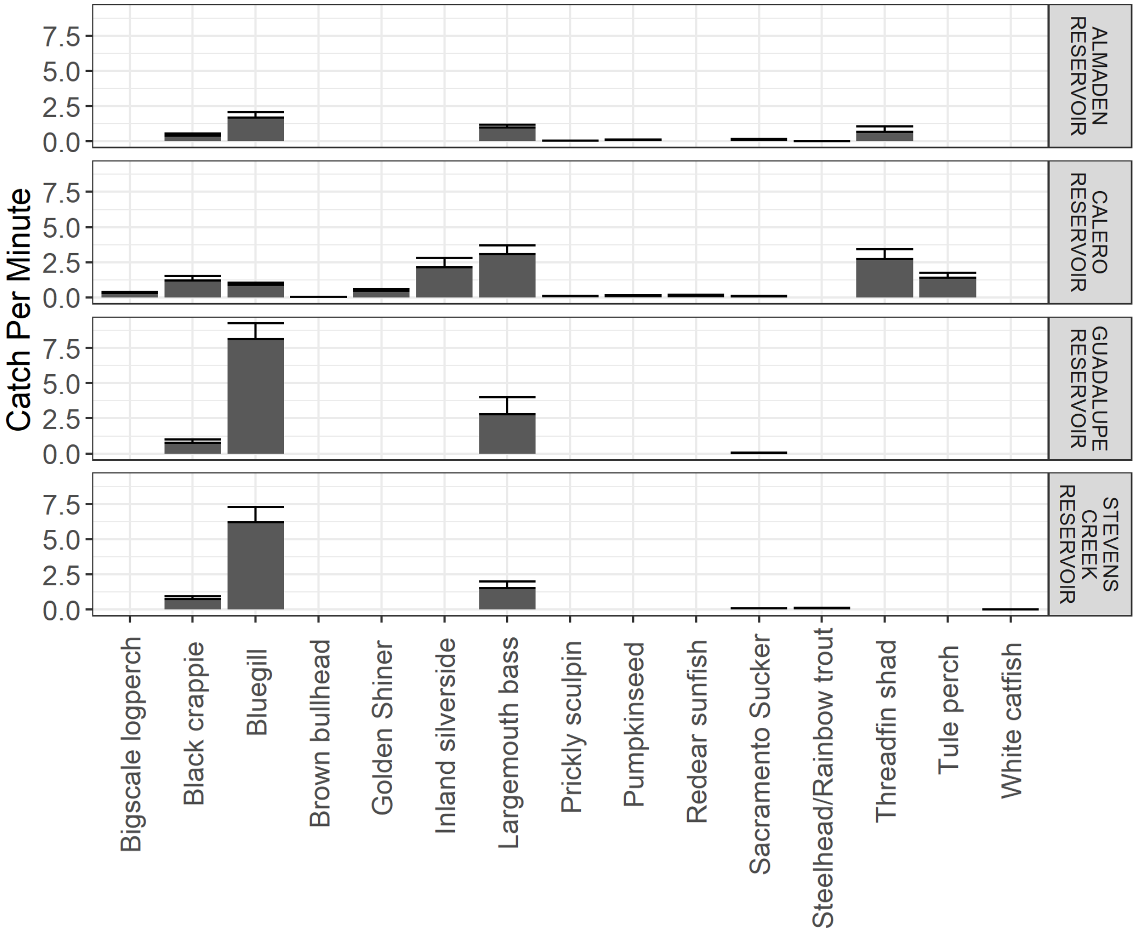

Figure S8. $\delta^{15}N$ vs. % MeHg (MeHg/Hg*100%) in SPM samples collected from surface waters. The blue line shows the least-squares linear regression, and the grey band is the 95% confidence interval. Isotope values are shown as the mean of triplicate subsamples.

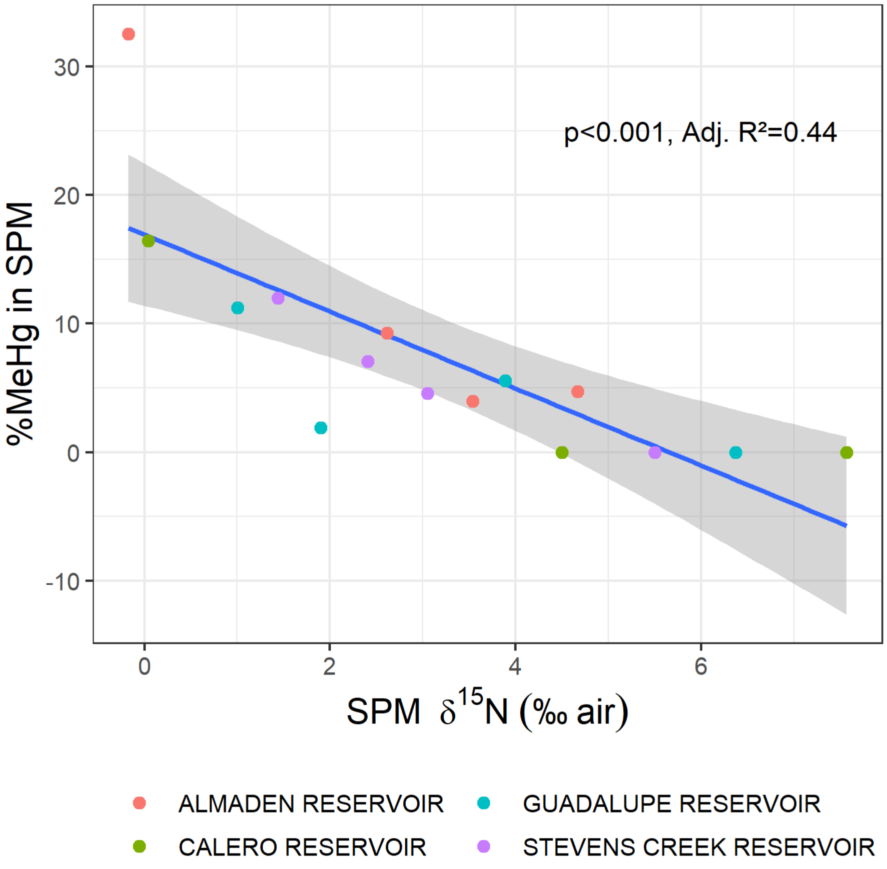

Figure S9. C:N ratios (top), $\delta^{13}C$ (middle), and $\delta^{15}N$ (bottom) measured in zooplankton composites from each reservoir.

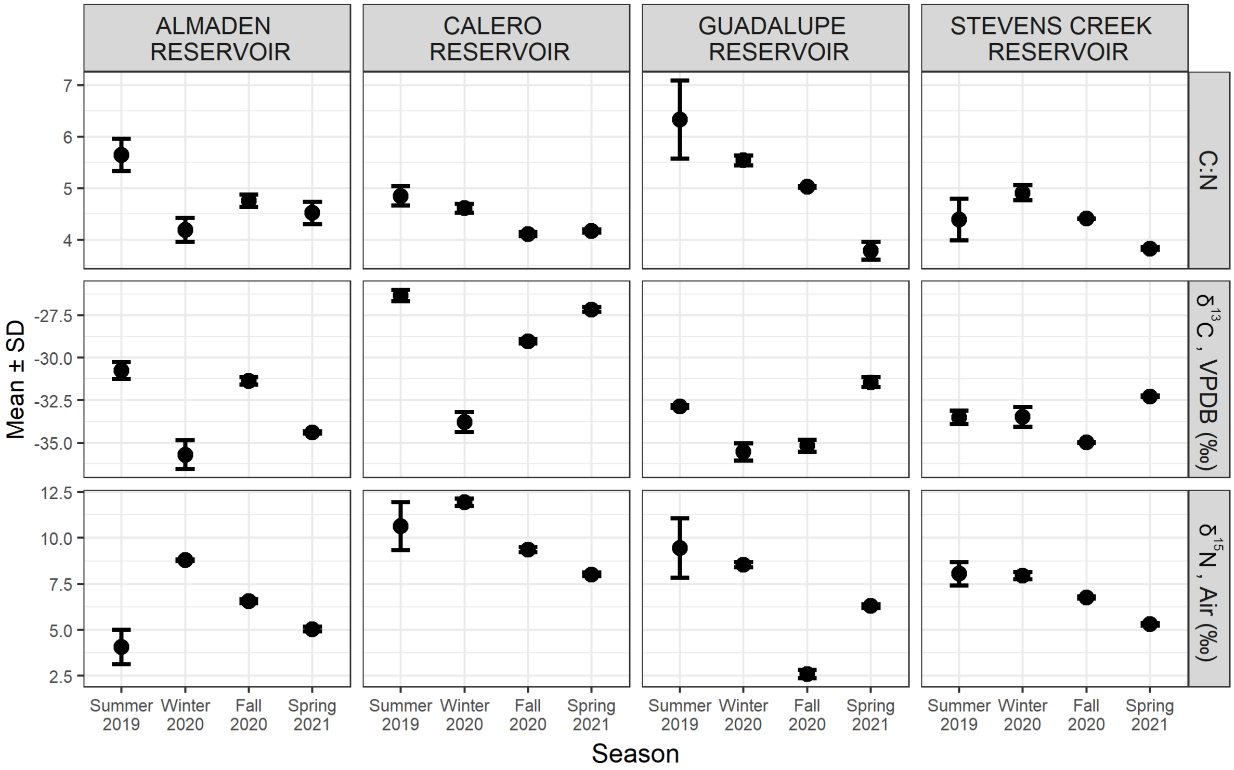

Figure S10. δ^{15}N in zooplankton vs. δ^{15}N in SPM measured at the surface (top), middle (middle), and bottom (bottom) sample depths. Isotope values are shown as mean ± 1 SD (n=3)

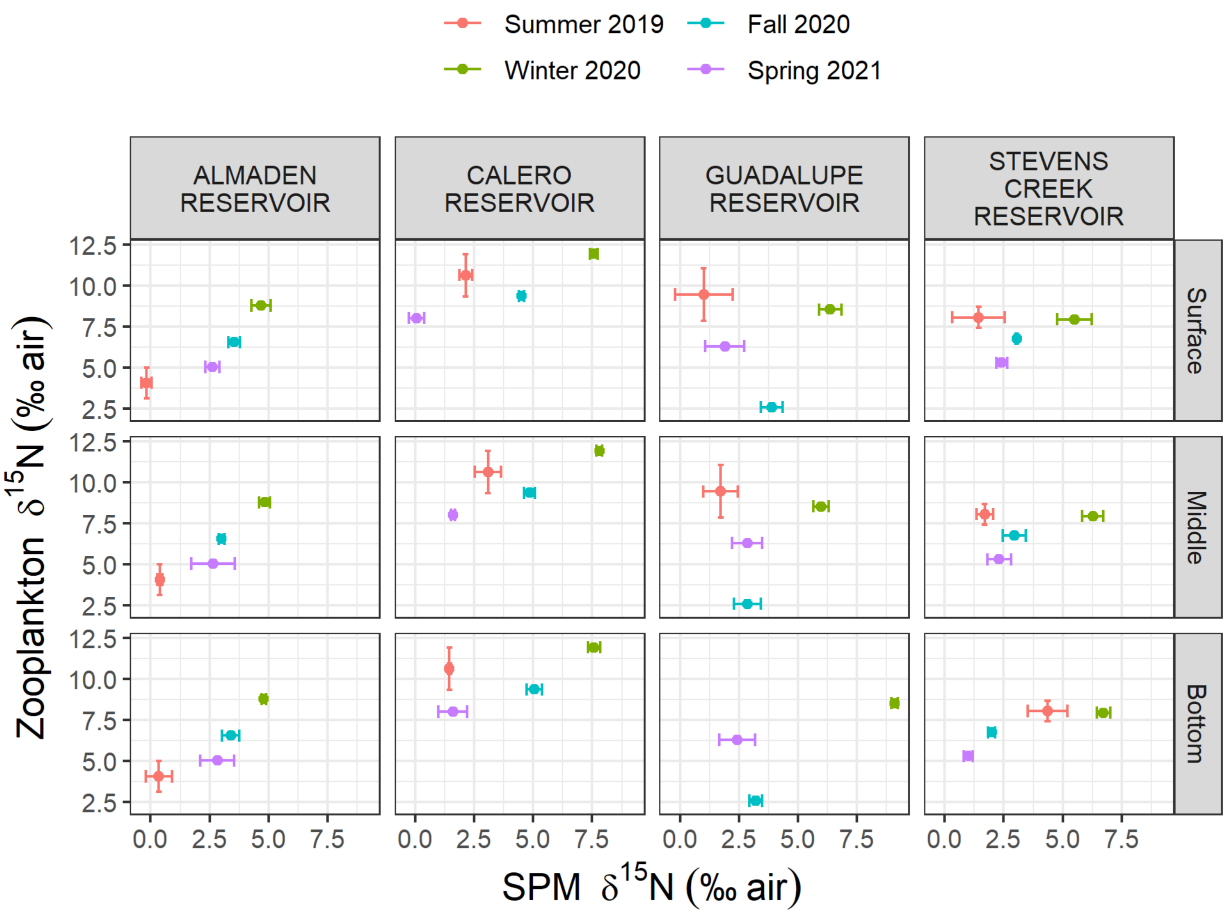

Figure S11. $\delta^{15}N$ in zooplankton composites vs. the mass percentage of copepods in the composite. The blue line shows the least-squares linear regression, and the grey band is the 95% confidence interval. Isotope values are shown as mean ± 1 SD (n=3).

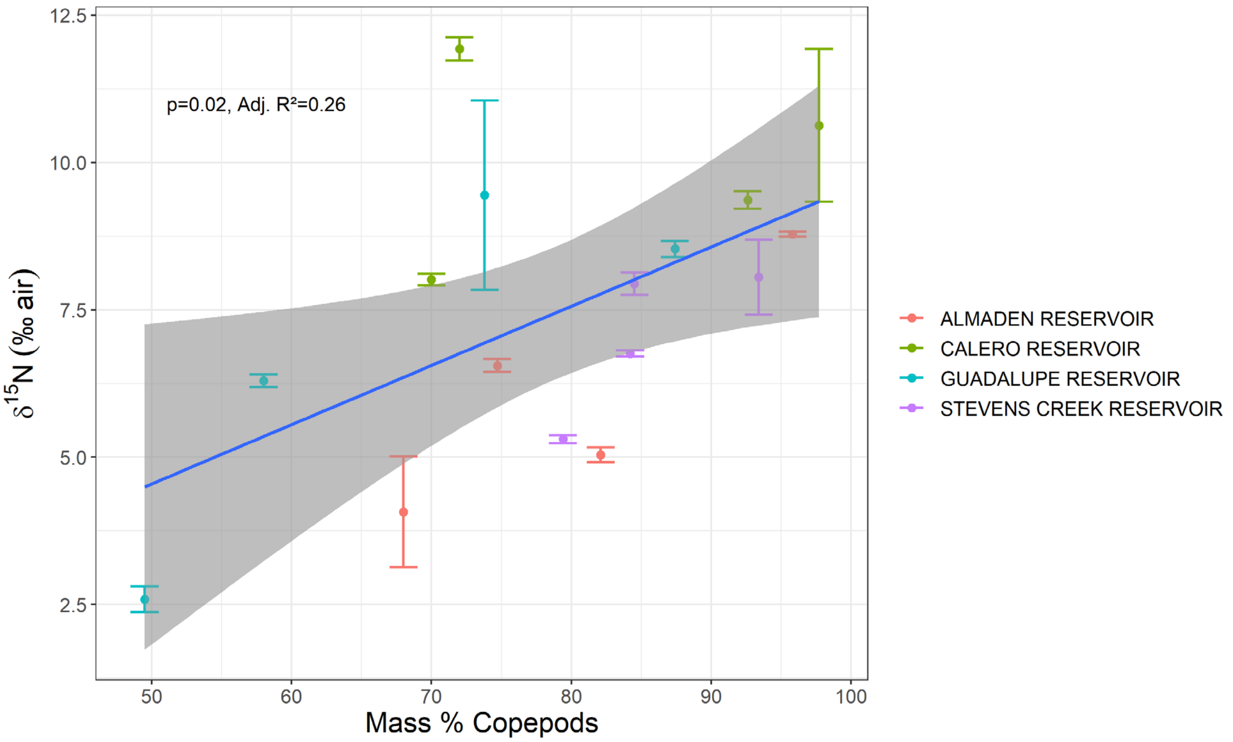

References

J. Dumont, G. Balvay. The dry weight estimate of Chaoborus flavicans (Meigen) as a function of length and instars. Hydrobiologia, Springer, 1979, 64 (2), pp.139-145. ⟨hal-02729002⟩

Ying Hong, Alan Steinman, Bopaiah Biddanda, Richard Rediske, Gary Fahnenstiel "Occurrence of the Toxin-producing Cyanobacterium Cylindrospermopsis raciborskii in Mona and Muskegon Lakes, Michigan," Journal of Great Lakes Research, 32(3), 645-652, (1 September 2006)

Jia, Nannan & Yang, Yiming & Yu, Gongliang & Wang, Yilang & Qiu, Pengfei & Li, Hua & Li, Renhui. (2020). Interspecific competition reveals Raphidiopsis raciborskii as a more successful invader than Microcystis aeruginosa. Harmful Algae. 97. 101858. 10.1016/j.hal.2020.101858. Matthews 2016

Olenina, Irina & Hajdu, Susanna & Edler, L. & Andersson, Agneta & Wasmund, N. & Busch, S. & Göbel, Jeanette & Gromisz, Sławomira & Huseby, S. & Huttunen, M. & Jaanus, Andres & Kokkonen, P. & Jurgensone, Iveta & Niemkiewicz, E.. (2006). Biovolumes and size-classes of phytoplankton in the Baltic Sea. HELCOM Balt. Sea Environ. Proc.. 106.

Wetzel, Robert G. and Gene E. Likens. Limnological Analysis Second Edition. New York: Springer, 1991. Print.
